# Countercation Manipulation Enables Selective Electrochemical CO_2_‐to‐Formate Conversion Over Bi‐Polyoxometalate‐Derived Catalysts

**DOI:** 10.1002/chem.71437

**Published:** 2026-07-22

**Authors:** Kimitake Kawakami, Kentaro Yonesato, Shimon Akino, Soichi Kikkawa, Seiji Yamazoe, Takuma Kaneko, Tomoya Uruga, Fumiaki Amano, Yoshiaki Honjo, Takaki Hatsui, Koji Harano, Kazuya Yamaguchi, Kosuke Suzuki

**Affiliations:** ^1^ Department of Applied Chemistry School of Engineering The University of Tokyo Tokyo Japan; ^2^ Department of Advanced Materials Science Graduate School of Frontier Sciences The University of Tokyo Kashiwa‐shi Chiba Japan; ^3^ Department of Chemistry Graduate School of Science Tokyo Metropolitan University Tokyo Japan; ^4^ Japan Synchrotron Radiation Research Institute (JASRI) Hyogo Japan; ^5^ Department of Applied Chemistry for Environment Graduate School of Urban Environmental Sciences Tokyo Metropolitan University Tokyo Japan; ^6^ RIKEN SPring‐8 Center Hyogo Japan; ^7^ Center for Basic Research on Materials National Institute for Materials Science (NIMS) Tsukuba‐shi Ibaraki Japan; ^8^ Research Center for Autonomous Systems Materialogy (ASMat) Institute of Integrated Research Institute of Science Tokyo Yokohama‐shi Kanagawa Japan

**Keywords:** bismuth, electrochemical CO_2_ reduction reaction, electrode catalysts, nanocatalysts, polyoxometalates

## Abstract

Electrochemical conversion of CO_2_ into formate (HCOO^−^) represents a promising strategy for mitigating global warming while producing an industrially important chemical. Polyoxometalates (POMs), anionic molecular metal‐oxo clusters, offer unique opportunities as catalysts and catalyst precursors for the electrochemical CO_2_ reduction reaction (CO_2_RR) owing to their structural diversity, compositional tunability, and flexibility in countercation selection. Despite these advantages, effective strategies for developing POM‐based CO_2_RR catalysts with high selectivity toward HCOO^−^ remain limited. In this study, we demonstrate countercation manipulation as a powerful approach to achieving selective HCOO^−^ production using a bismuth (Bi)‐incorporated POM, [Bi_4_O(γ‐SiW_10_O_36_)_2_(OAc)]^7−^ (**Bi4**). Under gas‐diffusion CO_2_RR conditions with a 2 M KHCO_3_ aqueous electrolyte, the Ba^2+^ salt of **Bi4** immobilized on carbon (Ba‐**Bi4**/C) exhibited high HCOO^−^ selectivity with a Faradaic efficiency of 84.3% at −0.79 V_RHE_, whereas the corresponding tetra‐*n*‐butylammonium (TBA^+^) salt (TBA‐**Bi4**/C) exhibited substantially lower selectivity (26.6% at −0.79 V_RHE_). Post‐reaction analyses revealed that both catalysts transformed into highly dispersed Bi species protected by WO*_x_* nanoaggregates. Control experiments and detailed characterizations indicated that the countercations, in conjunction with the POM‐derived catalyst structure, play a crucial role in governing CO_2_RR performance.

## Graphical abstract

Ba^2+^ salt of Bi‐incorporated polyoxometalate (POM), **Bi4** ([Bi_4_O(γ‐SiW_10_O_36_)_2_(OAc)]^7−^), immobilized on a carbon support (Ba‐**Bi4**/C) functioned as a formate (HCOO^−^)‐selective catalyst for electrochemical CO_2_ reduction reaction (CO_2_RR). The incorporation of Ba^2+^ and the formation of highly dispersed Bi species were the key factors for Ba‐**Bi4**/C to achieve efficient HCOO^−^ production.

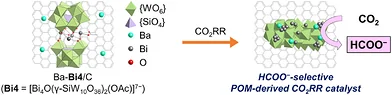

## 1 Introduction

Recent global warming, caused primarily by anthropogenic emissions of carbon dioxide (CO_2_), poses a serious threat to modern society [1]. To mitigate this climate crisis, considerable efforts have been devoted to developing technologies that utilize CO_2_ as a chemical feedstock. Among the various CO_2_ utilization strategies, electrochemical CO_2_ reduction reaction (CO_2_RR) is particularly attractive because it enables the conversion of CO_2_ into value‐added chemicals and fuels under mild conditions using renewable electricity [2, 3]. Among the possible CO_2_RR products, formic acid (HCOOH) and its conjugate base, formate (HCOO^−^), are especially appealing. HCOOH is an important industrial chemical widely used in agriculture, leather processing, food preservation, and pharmaceutical industries [4], and has recently attracted attention as a promising hydrogen carrier owing to its high stability, low toxicity, low flammability, and moderate volumetric energy density. Notably, the decomposition of HCOOH/HCOO^−^ produces only CO_2_ as a by‐product, enabling a closed carbon cycle in which CO_2_ can be recycled to regenerate HCOOH—a feature that is often difficult to achieve with other hydrogen carriers [5]. In addition, techno‐economic analyses suggest that CO_2_RR‐based production of HCOOH is considered economically viable [6]. Therefore, the development of efficient CO_2_RR systems for HCOOH/HCOO^−^ production is highly desirable.

The catalytic performance of CO_2_RR is strongly governed by both the catalyst material and the electrolyte composition. Among various metals, bismuth (Bi)‐based catalysts are known to exhibit high selectivity toward HCOO^−^ while effectively suppressing the competing hydrogen (H_2_) evolution reaction owing to their high overpotential for H_2_ production [7, 8]. In parallel, the identity of electrolyte cations plays a crucial role in determining CO_2_RR activity and selectivity. Larger alkali metal cations, such as Cs^+^ and K^+^, have been reported to enhance CO_2_RR performance by modulating the interfacial electric field, local pH, and stabilization of key reaction intermediates [9, 10, 11, 12, 13, 14, 15, 16, 17, 18, 19, 20]. Although the effects of alkali metal cations have been extensively studied, considerably fewer investigations have focused on multivalent cations, such as alkaline earth metal cations [13, 15, 17, 20, 21], despite both experimental and theoretical indications that Ba^2+^ can outperform smaller divalent cations such as Ca^2+^ [13, 17, 20, 21]. However, the use of alkaline earth metal cations at high concentration in CO_2_RR electrolytes—particularly in gas‐diffusion electrode systems—remains challenging, as the formation of insoluble carbonate or hydroxide species can hinder CO_2_ transport to the catalyst surface [15]. These insights suggest that combining Bi‐based catalysts with cations possessing large ionic radii represents a promising approach for selective CO_2_RR. Recently, Chang and coworkers investigated cation effects using Bi nanosheets and demonstrated that larger cations, such as Cs^+^ and Ba^2+^, enhance HCOOH productivity and selectivity in acidic electrolytes (pH 2) [20]. Nevertheless, a fundamental understanding of cation effects on Bi‐based CO_2_RR catalysts remains limited, and strategies to systematically exploit these effects are still at an early stage.

Polyoxometalates (POMs), anionic molecular metal‐oxo clusters composed of multiple {MO*_x_*} units (M = W, Mo, V, etc.), are characterized by their structural diversity, compositional tunability, and flexibility in countercation selection, as well as unique physicochemical properties such as acid‐base property and multielectron redox property [22, 23, 24, 25, 26, 27, 28, 29, 30, 31, 32, 33, 34, 35]. In particular, metal‐incorporated POMs enable atomic‐level control over active metal species and have emerged as promising (pre)catalysts for CO_2_RR as well as components of CO_2_RR catalysts [36, 37, 38, 39]. Our group has recently demonstrated that the catalytic performance of POM‐derived CO_2_RR catalysts is highly sensitive to the identity of countercations, with Ba^2+^ significantly enhancing activity and selectivity for carbon monoxide (CO) production [40, 41]. Despite these advances, POM‐based CO_2_RR catalysts that efficiently produce HCOOH/HCOO^−^ remain scarcely explored (Tables 1 and S1) [42, 43, 44, 45].

**TABLE 1:** Summary of POM‐based CO_2_RR catalysts exhibiting large FE_HCOOH/HCOO−_ (> 50%).

Catalyst	Electrolyte	Potential	FE_HCOOH/HCOO−_ (%)	*j*_HCOOH/HCOO−_ (mA cm^−2^)	Reference
Ba‐**Bi4**/C	2 M KHCO_3_	−0.79 V_RHE_	84.3	33.5	This work
		−0.87 V_RHE_	68.3	63.9	
Bi‐PMo nanosheets	0.5 M NaHCO_3_	−0.86 V_RHE_	93 ± 2	ca. 31	[42]
		−1.16 V_RHE_	ca. 67	ca. 39	
Indium sheet	0.1 M Na_2_SO_4_ containing 2 mM SiW_9_V_3_	−1.2 V_Ag/AgCl_ (−0.76 V_RHE_)	<5	<0.2	[43]
		−1.6 V_Ag/AgCl_	56.82	ca. 1.9	
		−1.7 V_Ag/AgCl_	ca. 42	ca. 2.6	
Cu_9_S_5_ single‐unit‐cell nanowires^a^	0.1 M KHCO_3_	−0.8 V_RHE_	82.0	*ca*. 2.5	[44]
		−0.9 V_RHE_	57.1	3.20	
TBA_3_H[(Ru(dmbpy)(ppt)Cl)_2_P_2_W_17_O_57_]^b^	0.1 M TBAPF_6_‐acetonitrile containing acetic acid	−1.56 V_NHE_	67	—	[45]

^a^ Nanowires composed of Cu_9_S_5_ unit cell and **PW12**.

^b^ dmbpy = 4,4'‐dimethyl‐2,2'‐dipyridyl, ppt = 4'‐(4‐phosphonophenyl)‐2,2':6',2''‐terpyridine.

In this work, we demonstrate that the rational combination of a Bi‐incorporated POM and a Ba^2+^ countercation provides an effective strategy for constructing HCOO^−^‐selective CO_2_RR catalysts (Figure 1). A Bi‐incorporated POM, **Bi4** ([Bi_4_O(γ‐SiW_10_O_36_)_2_(OAc)]^7−^, Figure 1a) [46], was synthesized by incorporating Bi^3+^ ions into a divacant lacunary POM, **SiW10** ([γ‐SiW_10_O_34_(H_2_O)_2_]^4−^) and subsequently immobilized on a carbon support (Figure 1b). The effect of countercation identity was investigated by comparing tetra‐*n*‐butylammonium (TBA^+^), Cs^+^, Mg^2+^, and Ba^2+^ salts. CO_2_RR experiments using a gas‐fed electrolyzer revealed that the Ba^2+^‐containing catalyst exhibited markedly enhanced HCOO^−^ production compared with its TBA^+^ analogue (Figure 1b). Through a combination of electrochemical analyses, x‐ray absorption fine structure analyses, and electron microscopy, we show that the Bi‐incorporated POM serves as a molecular precursor that undergoes in situ transformation under CO_2_RR conditions to generate highly dispersed Bi species stabilized by WO*_x_* nanoaggregates, while the Ba^2+^ countercation plays a crucial role in directing the reaction selectivity. This study establishes countercation manipulation as a powerful approach for achieving efficient and selective electrochemical HCOO^−^ production.

**FIGURE 1:**
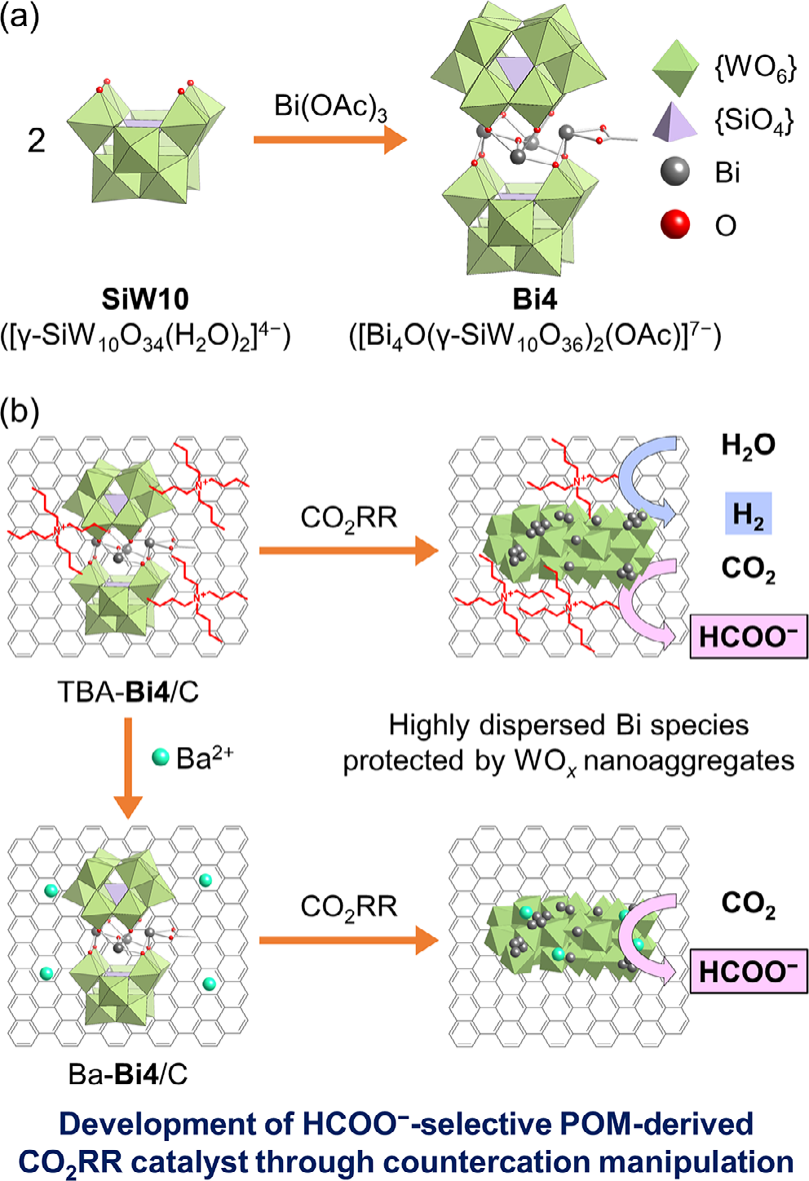
Preparation of POM‐derived Bi catalysts and their application in CO_2_RR. (a) Synthetic method and the structure of **Bi4** ([Bi_4_O(γ‐SiW_10_O_36_)_2_(OAc)]^7−^). (b) Schematic illustration of the catalyst preparation and CO_2_RR using TBA‐**Bi4**/C and Ba‐**Bi4**/C.

## 2 Results and Discussion

### 2.1 Preparation and Characterization of the Catalysts

Carbon‐supported Bi‐incorporated POM catalysts were prepared by adapting a previously reported impregnation method for POM‐derived electrocatalysts [41]. TBA‐**Bi4** (Figure 1a) [46] was first immobilized on a carbon support (Vulcan XC 72R), affording TBA‐**Bi4**/C (Figure 1b). Subsequently, the countercation of **Bi4** was exchanged from TBA^+^ to Ba^2+^, Mg^2+^, or Cs^+^ by vigorously stirring TBA‐**Bi4**/C in an acetone solution containing trifluoromethanesulfonate salts of Ba^2+^, Mg^2+^, or Cs^+^, yielding Ba‐**Bi4**/C, Mg‐**Bi4**/C, or Cs‐**Bi4**/C, respectively (Figure 1b).

To investigate the structure of **Bi4** after immobilization and cation exchange, Bi and W L_3_‐edge x‐ray absorption fine structure (XAFS) measurements were performed (Figure 2). At the Bi L_3_‐edge, both TBA‐**Bi4**/C and Ba‐**Bi4**/C exhibited extended x‐ray absorption fine structure (EXAFS) oscillation patterns similar to that of TBA‐**Bi4**, indicating preservation of the Bi coordination environment (Figure 2a). Notably, the Fourier‐transformed EXAFS spectrum of TBA‐**Bi4**/C displayed an increased peak intensity at approximately 1.7 Å (Bi─O bond) relative to that of TBA‐**Bi4**, whereas Ba‐**Bi4**/C exhibited a slightly lower peak intensity than TBA‐**Bi4**/C (Figure 2b). Similarly, at the W L_3_‐edge, the EXAFS oscillation patterns of TBA‐**Bi4**/C and Ba‐**Bi4**/C closely resembled those of TBA‐**Bi4** (Figure 2c). While TBA‐**Bi4** and TBA‐**Bi4**/C showed similar Fourier‐transformed EXAFS spectra, Ba‐**Bi4**/C showed a higher intensity for the peak at approximately 1.3 Å (terminal W═O bond) [47] and a lower intensity for the peak at approximately 1.7 Å (W─O(─W) bond) [47] compared with those of TBA‐**Bi4**/C (Figure 2d). These results indicated that immobilization and cation exchange induce only minor perturbations in the local coordination environment of Bi and W, while the overall **Bi4** structure remains largely intact in both TBA‐**Bi4**/C and Ba‐**Bi4**/C.

**FIGURE 2:**
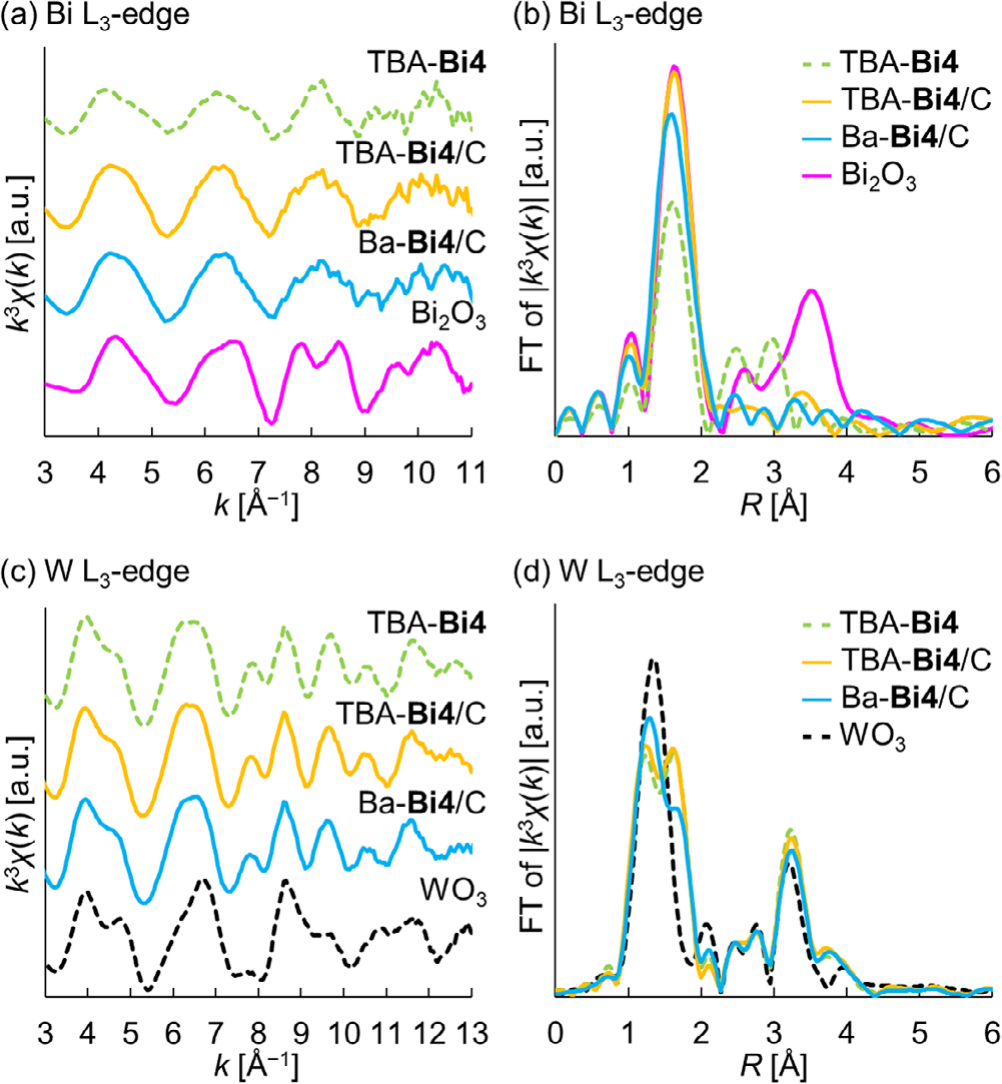
XAFS measurements of TBA‐**Bi4**/C and Ba‐**Bi4**/C. (a) *k* ^3^‐weighted Bi L_3_‐edge EXAFS oscillation. (b) Fourier‐transformed Bi L_3_‐edge EXAFS spectra (*k* = 3–11 Å^−1^). (c) *k* ^3^‐weighted W L_3_‐edge EXAFS oscillation. (d) Fourier‐transformed W L_3_‐edge EXAFS spectra (*k* = 3–13 Å^−1^).

Infrared (IR) spectroscopy was further employed to assess the structural integrity of **Bi4** in TBA‐**Bi4**/C and Ba‐**Bi4**/C. However, due to strong absorption from the carbon support, the characteristic IR absorption bands of **Bi4** could not be observed for TBA‐**Bi4**/C and Ba‐**Bi4**/C. Therefore, IR spectra of unsupported TBA‐**Bi4** and Ba‐**Bi4** were measured. As shown in Figure S1, Ba‐**Bi4** exhibited characteristic absorption bands in the range of 300–1000 cm^−1^ that closely resemble those of TBA‐**Bi4**. Importantly, no bands attributable to TBA^+^ were observed for Ba‐**Bi4** in the range of 1350–1500 cm^−1^, indicating successful countercation exchange without significant structural change to **Bi4**. Because Ba‐**Bi4** was prepared using the same protocol as Ba‐**Bi4**/C, these results support effective replacement of TBA^+^ with Ba^2+^ in the supported catalyst.

Elemental analysis further confirmed these findings (Table S2). Both TBA‐**Bi4**/C and Ba‐**Bi4**/C exhibited Bi:W ratios close to 4:20, consistent with the molecular composition of **Bi4** ([Bi_4_O(γ‐SiW_10_O_36_)_2_(OAc)]^7−^). Moreover, Ba‐**Bi4**/C showed a Ba:W ratio of 3.5:20. Considering the heptaanionic charge of **Bi4** and the divalent charge of Ba^2+^, this ratio supports nearly complete exchange of TBA^+^ with Ba^2+^.

### 2.2 CO_2_RR Study

The CO_2_RR performance of the catalysts was evaluated using a gas‐fed electrolyzer equipped with a gas‐diffusion electrode (Figure S2). In addition to the **Bi4**‐derived catalysts, a Ba^2+^ salt of **SiW10** ([γ‐SiW_10_O_34_(H_2_O)_2_]^4−^, a precursor of **Bi4**) supported on carbon (Ba‐**SiW10**/C) was prepared as a control sample (see Figure S3 and Table S2 for characterization). The catalyst‐loaded electrodes were fabricated by drop‐casting catalyst inks onto carbon fiber‐based gas‐diffusion layer (Sigracet 39 BB, see Supporting Information for detailed methods).

CO_2_ electrolysis was conducted at approximately −0.8 V_RHE_ (*iR*‐corrected) for 1 h under 80 mL min^−1^ CO_2_ flow with 2 M KHCO_3_ aqueous solution. Gaseous products (CO and H_2_) and the water‐soluble product (HCOO^−^) in the catholyte were quantified by gas chromatography and ^1^H NMR, respectively. As summarized in Figures 3a and S4a, and Table S3, Ba‐**Bi4**/C exhibited the highest partial current density (*j*_HCOO−_ = 33.5 mA cm^−2^) and Faradaic efficiency (FE_HCOO−_ = 84.3%) for HCOO^−^ production among all catalysts tested. No CO or HCOO^−^ was detected when the bare carbon support was used or when electrolysis was performed under an Ar atmosphere using Ba‐**Bi4**/C (Table S4), confirming that these products originate from CO_2_RR. Notably, Ba‐**Bi4**/C exhibited a *j*_HCOO−_value comparable to or higher than those of previously reported POM‐based CO_2_RR catalysts at similar applied potentials (Table 1). In contrast, Mg‐**Bi4**/C, Cs‐**Bi4**/C, and TBA‐**Bi4**/C exhibited lower CO_2_RR performance, with TBA‐**Bi4**/C predominantly producing H_2_ and showing significantly lower HCOO^−^ productivity and selectivity (*j*_HCOO−_ = 9.6 mA cm^−2^, FE_HCOO−_ = 26.6%), highlighting the importance of selecting appropriate countercations to achieve superior CO_2_RR performance. Bismuth(III) acetate (Bi(OAc)_3_), a precursor of **Bi4**, exhibited an even lower *j*_HCOO−_ value of 6.1 mA cm^−2^, although its FE_HCOO−_ was moderate (70.4%) (Figures 3b and S4b and Table S3). In addition, a physical mixture of Bi(OAc)_3_ and Ba‐**SiW10**/C (Ba‐**SiW10**/C+Bi(OAc)_3_) showed low CO_2_RR performance. We also evaluated the CO_2_RR performance of monolayer bismuth tungstate (Bi_2_WO_6_), known for efficiently producing HCOO^−^ [48], and bulk Bi_2_WO_6_ [49], but both samples exhibited substantially lower productivity and selectivity for HCOO^−^ production (Figures 3b and S4b and Table S3). These results underscore the critical role of Bi incorporation into the POM framework in achieving high HCOO^−^‐selective CO_2_RR performance.

**FIGURE 3:**
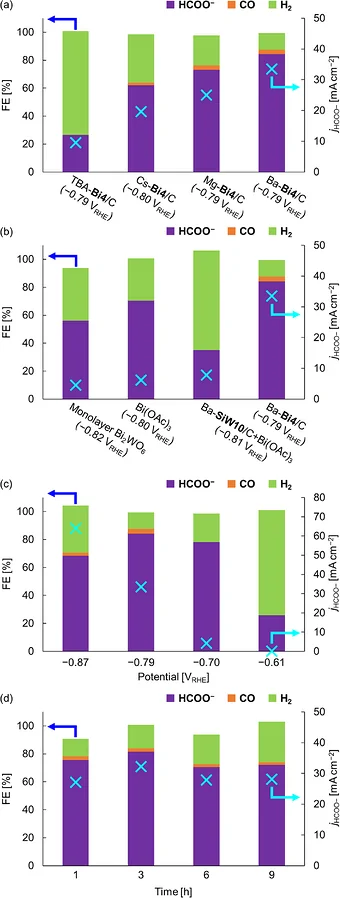
CO_2_RR performance of POM‐derived catalyst‐loaded electrodes with 2 M KHCO_3_ flow: FE (left axis) and *j*_HCOO−_ (right axis). (a) **Bi4**/C possessing various countercations at a constant potential around −0.8 V_RHE_ for 1 h. (b) Ba‐**Bi4**/C and other benchmark catalysts at a constant potential around −0.8 V_RHE_ for 1 h. (c) Ba‐**Bi4**/C at different potentials for 1 h. (d) Ba‐**Bi4**/C at −0.82 V_RHE_ for 9 h. All potentials were *iR*‐corrected.

Potential‐dependent measurements revealed a volcano‐type relationship between HCOO^−^ selectivity and the applied potential for Ba‐**Bi4**/C (Figures 3c and S4c and Table S5), with a maximum FE_HCOO−_ of 84.3% observed at −0.79 V_RHE_. At more negative potentials, the *j*_HCOO−_ increased further, reaching 63.9 mA cm^−2^ at −0.87 V_RHE_, representing the highest value reported to date for POM‐based CO_2_RR catalysts (Table 1). Tafel analysis revealed that the Tafel slope values in the low overpotential region were 55, 54, 63, and 66 mV dec^−1^ for Ba‐**Bi4**/C, TBA‐**Bi4**/C, Ba‐**SiW10**/C+Bi(OAc)_3_, and Bi(OAc)_3_, respectively (Figure S5). Notably, these values are close to the theoretical value of 59 mV dec^−1^, indicating that the protonation of *CO_2_^−^ to form *OCHO is the rate‐limiting step across these samples [42, 50, 51, 52]. This kinetic similarity suggested that the enhanced HCOO^−^ productivity of Ba‐**Bi4**/C cannot be attributed to a change in the rate‐limiting step but to other factors (as discussed in detail in the following sections). Subsequently, long‐term CO_2_RR electrolysis was conducted at −0.82 V_RHE_ for 9 h using Ba‐**Bi4**/C (Figures 3d and S4d,e and Table S6). Although the selectivity to H_2_ gradually increased during the prolonged electrolysis, the FE_HCOO−_ remained above 70% even after 9 h, and the *j*_HCOO−_ remained nearly constant throughout the experiment. In addition, although the slight fluctuations were observed during the initial 4 h, the total current density stabilized at approximately 40 mA cm^−2^ thereafter, demonstrating the robust electrochemical stability of the catalyst.

### 2.3 Characterization of a Ba‐Bi4/C‐Loaded Electrode After CO_2_RR

Based on the above results, we next discuss the structural evolution of Ba‐**Bi4**/C under CO_2_RR conditions.

We first performed XAFS measurements on Ba‐**Bi4**/C electrodes before and after immersion in the catholyte (2 M KHCO_3_) for 1 h to examine the effect of catholyte contact on the electronic state and the structure of **Bi4**. As displayed in Figure 4, both electrodes exhibited x‐ray absorption near edge structure (XANES) spectra (Figure 4a) and EXAFS oscillation patterns that closely resemble those of pristine Ba‐**Bi4**/C (Figure 4b,d), although slight variations in the peak intensities associated with the Bi─O bond (peak around 1.7 Å, Figure 4c), the terminal W═O bond (peak around 1.3 Å, Figure 4e) [47], and the W─O(─W) bond (peak around 1.7 Å, Figure 4e) [47] were observed. These results suggest that, although minor perturbations occurred during electrode fabrication and catholyte contact, the overall electronic state and the molecular structure of **Bi4** were largely retained.

**FIGURE 4:**
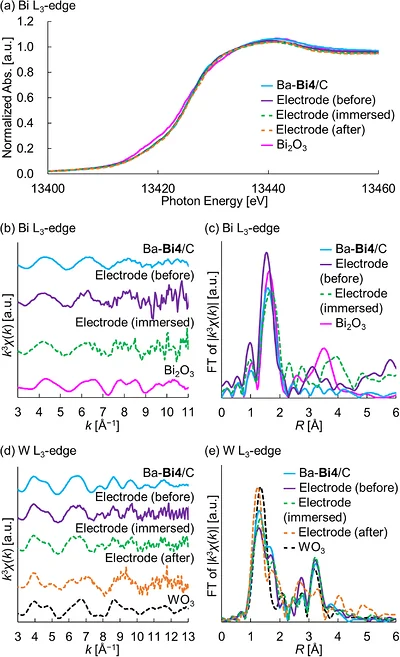
XAFS measurements of Ba‐**Bi4**/C‐loaded electrodes. (a) Bi L_3_‐edge XANES spectra. (b) *k* ^3^‐weighted Bi L_3_‐edge EXAFS oscillation. (c) Fourier‐transformed Bi L_3_‐edge EXAFS spectra (*k* = 3–11 Å^−1^). (d) *k* ^3^‐weighted W L_3_‐edge EXAFS oscillation. (e) Fourier‐transformed W L_3_‐edge EXAFS spectra (*k* = 3–13 Å^−1^).

To identify the catalytically active Bi species generated under CO_2_RR conditions, cyclic voltammetry (CV) of the Ba‐**Bi4**/C electrode after CO_2_RR at −0.8 V_RHE_ for 1 h was conducted (Figure S6). A single cathodic peak appeared in the range of 0.1–0.2 V_RHE_, which can be assigned to the reduction of Bi^3+^ to Bi^0^ [53]. Given that CO_2_RR was conducted at much more negative potentials (approximately −0.8 V_RHE_), Bi^0^ species were likely generated during electrolysis and served as the truly catalytically active sites.

Further insight into the post‐reaction catalyst structure was obtained by XAFS measurements of the Ba‐**Bi4**/C electrode after CO_2_RR (Figure 4). The Bi L_3_‐edge XANES spectrum of the post‐reaction electrode closely overlapped with those of pristine Ba‐**Bi4**/C and the electrode before the reaction, indicating that Bi was predominantly present as Bi^3+^ after the reaction (Figure 4a). This observation does not contradict the CV results, as the ex situ XAFS measurements were performed after exposure of the electrode to air, which can readily oxidize metallic Bi. At the W L_3_‐edge, the EXAFS oscillation pattern of the electrode after CO_2_RR more closely resembled that of WO_3_ than that of the electrode before the reaction; however, clear differences were observed in the *k* range of 7–9 Å^−1^ and in the Fourier‐transformed spectra around 3–4 Å (Figure 4d,e). These features suggest the formation of WO*_x_* species distinct from bulk WO_3_ during CO_2_RR.

To further elucidate the nanoscale structure of the catalyst after CO_2_RR, electron microscopy analysis was performed. We initially carried out atomic‐resolution transmission electron microscopy (TEM) observation of the Ba‐**Bi4**/C electrode after a 1 h CO_2_RR (Figure S7). However, the detailed analysis of catalyst morphology was difficult due to the low contrast between the carbon support and the catalyst. High‐angle annular dark‐field scanning transmission electron microscopy (HAADF‐STEM) image (Figure 5a) and the corresponding STEM energy‐dispersive X‐ray spectroscopy (EDS) mappings (Figure 5b–e) suggest the formation of WO*_x_* nanoaggregates within which Bi species are highly dispersed. Although some Bi species formed nanoparticles within WO*_x_* nanoaggregates (Figure S8), low‐magnification STEM images also verified that most of the Bi species were highly dispersed (Figure S9). Liu and coworkers reported that, under CO_2_RR conditions, monolayer bismuth tungstate (Bi_2_WO_6_) reconstructs to form metallic Bi stabilized by tungsten oxide species, as revealed by in situ characterization techniques [48]. In light of this report, it is reasonable to conclude that, in the present system, Bi species are likewise stabilized by WO*_x_* nanoaggregates.

**FIGURE 5:**
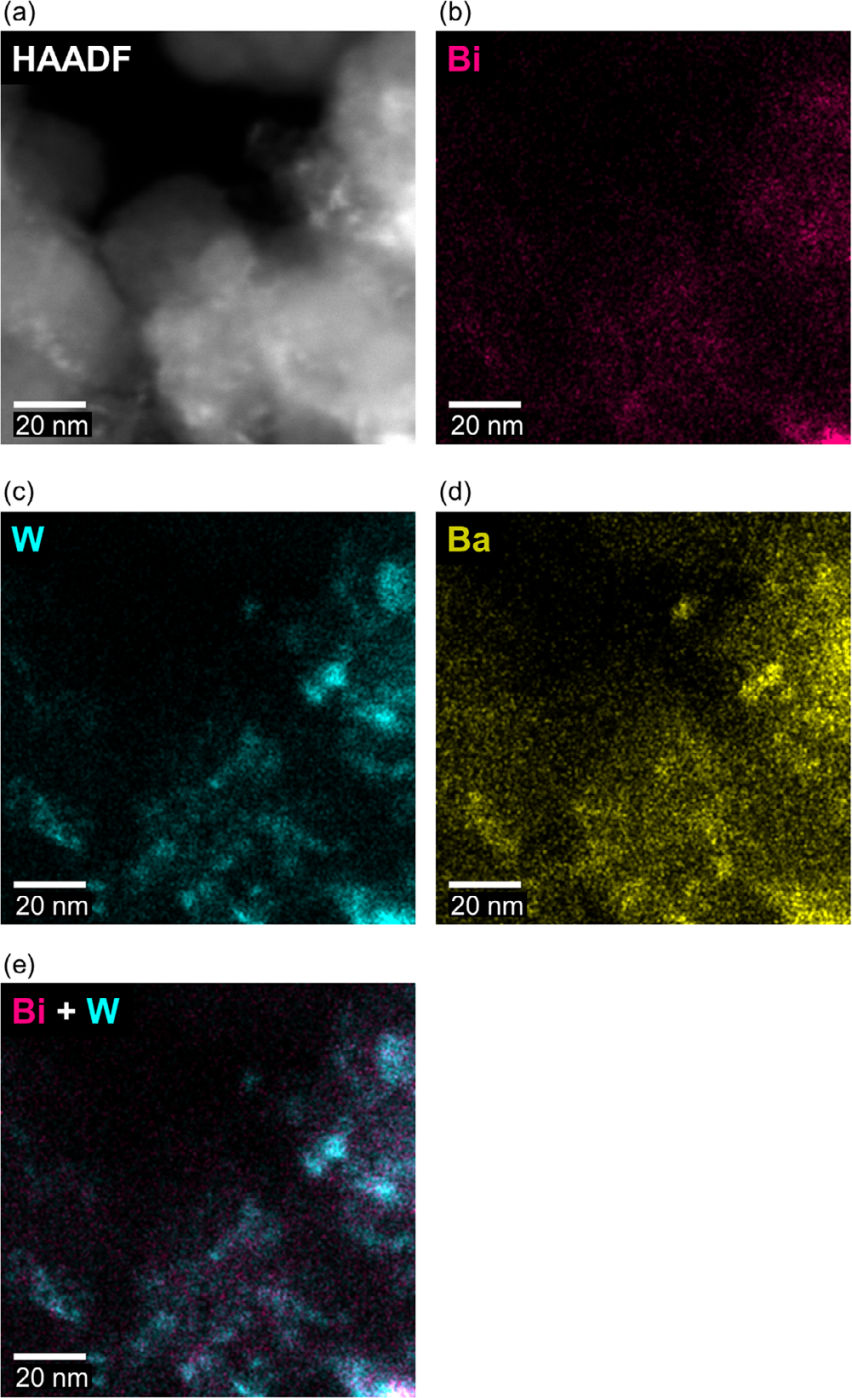
STEM images of Ba‐**Bi4**/C‐loaded electrode after a 1 h CO_2_RR. (a) HAADF‐STEM image and (b–e) STEM‐EDS mappings (Bi, magenta; W, cyan; Ba, yellow).

Taken together, the CV, XAFS, and STEM results demonstrate that Ba‐**Bi4**/C is transformed into highly dispersed Bi species protected by WO*_x_* nanoaggregates under CO_2_RR conditions.

### 2.4 Effect of Countercations on CO_2_RR Performance

As shown in the former section, TBA‐**Bi4**/C exhibited substantially lower CO_2_RR performance than Ba‐**Bi4**/C. In this section, we discuss the role of countercation in determining the CO_2_RR performance.

Post‐reaction characterization of the TBA‐**Bi4**/C electrode after CO_2_RR at −0.8 V_RHE_ was first conducted. CV measurement revealed a cathodic peak in the range of 0.1–0.2 V_RHE_ corresponding to the reduction of Bi^3+^ to Bi^0^, indicating that Bi^0^ is also generated as the active species under CO_2_RR conditions (Figure S6). XAFS analysis further clarified that the Bi L_3_‐edge XANES spectra and W L_3_‐edge EXAFS oscillation patterns of TBA‐**Bi4**/C and Ba‐**Bi4**/C electrodes after the reaction were largely comparable, with only minor differences observed in the Fourier‐transformed W L_3_‐edge EXAFS spectra (Figure S10). Consistent with these results, STEM analysis revealed the formation of highly dispersed Bi species protected by WO*_x_* nanoaggregates on the TBA‐**Bi4**/C electrode after the reaction (Figure S11). These observations suggest that both catalysts underwent similar structural transformations during CO_2_RR. Elemental analysis was performed to quantify the Bi content remaining on the electrodes after reaction. As summarized in Table S7, although initial Bi loadings were nearly identical for both samples, the Ba‐**Bi4**/C electrode after the reaction exhibited a slightly lower Bi loading than the TBA‐**Bi4**/C electrode after the reaction. This result suggests that differences in Bi loading are unlikely to be the primary origin of the large performance difference.

It has been reported that electron‐rich Bi‐based CO_2_RR catalysts tend to exhibit higher CO_2_RR performance, as electron‐rich Bi sites facilitate either the reduction of *OCHO intermediates or the formation of *CO_2_^−^ intermediates [54, 55]. To assess the electronic state of Bi, CV measurements were performed on TBA‐**Bi4**/C and Ba‐**Bi4**/C electrodes after the reaction (Figure S6). Notably, the reduction peak of Bi^3+^ for the TBA‐**Bi4**/C electrode appeared at a potential approximately 10 mV more negative than that for the Ba‐**Bi4**/C electrode. Chen and coworkers reported a correlation among the reduction potential of Bi^3+^, the electronic state of Bi, and the CO_2_RR performance, demonstrating that more electron‐rich Bi species exhibit more negative reduction potentials and higher CO_2_RR activity [54]. Accordingly, TBA‐**Bi4**/C appears to generate slightly more electron‐rich Bi species, consistent with our previous report [40]. This difference may originate from countercation‐dependent electron‐donating properties of the WO*_x_* nanoaggregates.

Importantly, despite possessing slightly more electron‐rich species, TBA‐**Bi4**/C exhibited inferior CO_2_RR performance. This finding indicates that electronic effects cannot account for the enhanced CO_2_RR performance of Ba‐**Bi4**/C. We therefore attribute the superior performance of Ba‐**Bi4**/C primarily to cation effects. Although the origin of cation effects in CO_2_RR remains under debate, three main contributions have been proposed: (a) modulation of the electric field near the electrode surface; (b) buffering of the local pH near the electrode surface; and (c) stabilization of reaction intermediates [56, 57, 58, 59]. While it is difficult to unambiguously identify the dominant factor in the present system, it is reasonable to conclude that Ba^2+^‐induced cation effects play a critical role in enhancing the CO_2_RR performance of Ba‐**Bi4**/C.

### 2.5 Effect of Catalyst Structure on CO_2_RR Performance

As described in the CO_2_RR Study Section, Bi(OAc)_3_ exhibited significantly lower CO_2_RR performance than Ba‐**Bi4**/C. In this section, we discuss the influence of the catalyst structure on CO_2_RR performance.

After CO_2_RR at −0.8 V_RHE_ for 1 h, CV analysis of the Bi(OAc)_3_‐derived electrode revealed a reduction peak corresponding to the conversion of Bi^3+^ to Bi^0^ in the range of 0.0–0.3 V_RHE_, indicating that Bi^0^ species also serve as the active species in this system (Figure S6). Ex situ XAFS measurement suggested that in situ‐generated Bi^0^ species were fully oxidized to Bi^3+^ after the reaction (Figure S12). TEM analysis further revealed the formation of Bi nanoparticles with an average diameter of 2.9 ± 1.2 nm after the reaction (Figure S13). These outcomes all suggest that Bi(OAc)_3_ is transformed into small Bi nanoparticles during the reaction. Elemental analysis (Table S7) showed that the Bi content remaining on the Bi(OAc)_3_ electrode after CO_2_RR was approximately twice that on the Ba‐**Bi4**/C electrode after the reaction, suggesting that the inferior CO_2_RR performance observed for Bi(OAc)_3_ may not stem from lower Bi loading.

CV measurements comparing the electronic states of Bi species formed on Bi(OAc)_3_ and Ba‐**Bi4**/C electrodes after reaction showed that the reduction peak for Ba‐**Bi4**/C shifted by approximately 10 mV to a more negative potential, suggesting the formation of slightly more electron‐rich Bi species (Figure S6). However, given the small magnitude of this shift, electronic effects are unlikely to be the dominant factor governing the observed performance difference. Instead, we attribute the superior performance of Ba‐**Bi4**/C primarily to its ability to generate and stabilize highly dispersed Bi species. The electrochemical impedance spectroscopy at −0.8 V_RHE_ revealed that the catalyst derived from Ba‐**Bi4**/C exhibited significantly lower charge transfer resistance than that derived from Bi(OAc)_3_ (Figure S14). The close proximity between Bi and {WO_6_} units in Ba‐**Bi4**/C facilitates the formation of isolated or highly dispersed Bi species stabilized by WO*_x_* nanoaggregates, whereas Bi(OAc)_3_, lacking such protection by WO*_x_* species, readily aggregates into Bi nanoparticles. As a consequence, Ba‐**Bi4**/C showed faster electron transfer kinetics and higher CO_2_RR performance.

## 3 Conclusion

In this study, we demonstrated that countercation manipulation provides a facile and powerful strategy for tuning the performance of POM‐based electrocatalysts for CO_2_RR. A bismuth (Bi)‐incorporated POM, [Bi_4_O(γ‐SiW_10_O_36_)_2_(OAc)]^7−^ (**Bi4**), was immobilized on a carbon support via an impregnation method to afford TBA‐**Bi4**/C, followed by countercation exchange with Ba^2+^ to yield Ba‐**Bi4**/C. When evaluated as gas‐diffusion electrodes in a gas‐fed CO_2_ electrolyzer using 2 M KHCO_3_ aqueous electrolyte, Ba‐**Bi4**/C exhibited markedly enhanced formate (HCOO^−^) production, achieving a high partial current density (*j*_HCOO−_ = 33.5 mA cm^−2^) and Faradaic efficiency (FE_HCOO−_ = 84.3%) at −0.79 V_RHE_. In contrast, TBA‐**Bi4**/C showed significantly lower HCOO^−^ selectivity (FE_HCOO−_ = 26.6% at −0.79 V_RHE_), while Bi(OAc)_3_, a precursor of **Bi4**, exhibited lower HCOO^−^ productivity (*j*_HCOO−_ = 6.1 mA cm^−2^). Post‐reaction analyses revealed that both Ba‐**Bi4**/C and TBA‐**Bi4**/C underwent in situ structural reconstruction during CO_2_RR to form highly dispersed Bi species stabilized by WO*_x_* nanoaggregates, whereas Bi(OAc)_3_ was transformed into aggregated Bi nanoparticles. Systematic control experiments and characterizations highlighted the critical roles of both countercation identity and molecular structure of the precatalyst in achieving high CO_2_RR performance, underscoring countercation manipulation as a key design parameter.

Given that HCOO^−^‐selective POM‐based CO_2_RR catalysts remain scarce and the role of cations in Bi‐based CO_2_RR catalysis is still not fully understood, this work provides new insights into cation effects and establishes a generalizable strategy for designing efficient POM‐based CO_2_RR catalysts. We anticipate that the concept of countercation engineering demonstrated here will inspire further development of molecularly defined electrocatalysts for selective and efficient CO_2_ conversion.

## Funding

Japan Science and Technology Agency. Japan Society for the Promotion of Science

## Conflicts of Interest

The authors declare no conflicts of interest.

## Supporting information




**Supporting File 1**: chem71437‐sup‐0001‐SuppMat.pdf The authors have cited additional references within the Supporting Information [60, 61, 62, 63, 64, 65, 66, 67, 68, 69, 70, 71, 72, 73, 74, 75, 76, 77, 78, 79, 80, 81, 82, 83, 84, 85, 86, 87, 88, 89, 90, 91, 92, 93, 94, 95, 96]. The Supporting Information includes experimental section and supplementary figures and tables.

## Data Availability

The data that support the findings of this study are available in the Supporting Information of this article.
