# Supplementary material for: Countercation Manipulation Enables Selective Electrochemical CO2‐to‐Formate Conversion Over Bi‐Polyoxometalate‐Derived Catalysts
Source: Chemistry. 2026 Jul 22;32(40):e71437. doi: 10.1002/chem.71437 (PMC13716004; doi:10.1002/chem.71437)
Supplement: Supplementary file 1 [file CHEM-32-e71437-s001.pdf]

Supporting Information for

Countercation Manipulation Enables Selective Electrochemical CO_2-to-Formate Conversion over Bi–Polyoxometalate-Derived Catalysts

Kimitake Kawakami,[a] Kentaro Yonesato,[ab] Shimon Akino,[a] Soichi Kikkawa,[c] Seiji Yamazoe,[c] Takuma Kaneko,[d] Tomoya Uruga,[d] Fumiaki Amano,[e] Yoshiaki Honjo,[f] Takaki Hatsui,[f] Koji Harano,[g,h] Kazuya Yamaguchi,[a] and Kosuke Suzuki*[ab]

[a] *Department of Applied Chemistry, School of Engineering, The University of Tokyo, 7-3-1 Hongo, Bunkyo-ku, Tokyo 113-8656, Japan.*

[b] *Department of Advanced Materials Science, Graduate School of Frontier Sciences, The University of Tokyo, 5-1-5 Kashiwa-no-ha, Kashiwa-shi, Chiba 277-8563, Japan. E-mail: ksuzuki@appchem.t.u-tokyo.ac.jp*

[c] *Department of Chemistry, Graduate School of Science, Tokyo Metropolitan University, 1-1 Minami-Osawa, Hachioji-shi, Tokyo 192-0397, Japan.*

[d] *Japan Synchrotron Radiation Research Institute (JASRI), 1-1-1 Kouto, Sayo-cho, Sayo-gun, Hyogo 679-5198, Japan.*

[e] *Department of Applied Chemistry for Environment, Graduate School of Urban Environmental Sciences, Tokyo Metropolitan University, 1-1 Minami-Osawa, Hachioji-shi, Tokyo 192-0397, Japan.*

[f] *RIKEN SPring-8 Center, RIKEN, 1-1-1 Kouto, Sayo-cho, Sayo-gun, Hyogo 679-5148, Japan.*

[g] *Center for Basic Research on Materials, National Institute for Materials Science (NIMS), 1-1 Namiki, Tsukuba-shi, Ibaraki 305-0044, Japan.*

[h] *Research Center for Autonomous Systems Materialogy (ASMat), Institute of Integrated Research, Institute of Science Tokyo, 4259 Nagatsuta-cho, Midori-ku, Yokohama-shi, Kanagawa 226-8501, Japan.*

Contents

		Page
1.	Experimental Section	S2
2.	Supplementary Figures	S6
3.	Supplementary Tables	S15
4.	References	S22

1. Experimental Section

Instruments

GC analyses were carried out on Nexis GC-2030 gas chromatography (Shimadzu Corporation) equipped with a barrier ionization discharge (BID) detector and a ShinCarbon ST Micropacked column (2.0 m × 1.0 mm I.D., Shinwa Chemical Industries Ltd.). Electrochemical measurements were conducted on VMP3 and VSP-300 multichannel potentiostats (BioLogic). ^{1}H NMR (500 MHz) measurements were performed on JNM ECA-500 and JNM-ECZ500R/S1 spectrometers (JEOL Ltd.) using 5-mm outer-diameter tubes. ^{1}H NMR chemical shifts were referenced to dimethyl sulfoxide signal (2.6 ppm).[1] IR measurements were carried out on FT/IR-4100 (JASCO Corporation) using KBr disks. ICP-AES measurements were conducted on ICP-8100 (Shimadzu Corporation). TEM observations were conducted on JEM-2010F (JEOL Ltd.). Atomic-resolution TEM observations were performed on JEM-ARM200F NEOARM (JEOL Ltd.) equipped with a RIO16 camera (Gatan Inc.) at an acceleration voltage of 80 kV. HAADF-STEM observations and STEM-EDS mappings were performed on Talos F200X G2 (Thermo Fisher Scientific, Inc.) equipped with an EDS detector (Super-X G2) at an acceleration voltage of 80 kV.

Materials

Acetone, acetonitrile, dimethyl sulfoxide, D_2O, phenol, potassium bicarbonate ($KHCO_3$), potassium hydroxide (KOH), and sodium chloride (NaCl) were obtained from Kanto Chemical Co., Inc. Barium trifluoromethanesulfonate ($Ba(OTf)_2$) was purchased from Strem Chemicals, Inc. Magnesium trifluoromethanesulfonate ($Mg(OTf)_2$) and 10% tetrabutylammonium hydroxide (TBAOH)-methanol solution were acquired from Tokyo Chemical Industry Co., Ltd. 5% Nafion dispersion was obtained from FUJIFILM Wako Pure Chemical Corporation. Bismuth(III) acetate ($Bi(OAc)_3$) was purchased from Sigma-Aldrich. A carbon black (Vulcan XC 72R) and cation exchange membranes (CEMs, HP110 ePTFE Reinforced PFSA Membrane) were acquired from Fuel Cell Store. CEMs were pre-activated by immersing CEMs in 1 M KOH aqueous solution, and then, in 0.1 M $KHCO_3$ aqueous solution for several days. Carbon fiber-based gas-diffusion layer (Sigracet 39 BB) was obtained from MFC Technology Inc. Argon (Ar) and carbon dioxide (CO_2, >99.995%) were purchased from TOMOE SHOKAI Co., LTD. TBA-**Bi4** ($TBA_7[Bi_4O(\gamma\text{-}SiW_{10}O_{36})_2(OAc)]\cdot 2CH_3COCH_3\cdot H_2O$, $TBA^+ = n\text{-}Bu_4N^+$), TBA-**SiW10** ($TBA_4[\gamma\text{-}SiW_{10}O_{34}(H_2O)_2]\cdot H_2O$)[3], cesium trifluoromethanesulfonate (CsOTf)[4], monolayer bismuth tungstate (Bi_2WO_6)[5], and bulk Bi_2WO_6[6] were synthesized according to the reported procedures.

Preparation of TBA-Bi4/C and TBA-SiW10/C

TBA-**Bi4**/C and TBA-**SiW10**/C were prepared by referring to the previous literature.[7] A carbon black support (Vulcan XC 72R, 120 mg) was dispersed in acetone (180 mL) with the aid of ultrasonication. While vigorously stirring the suspension, TBA-**Bi4** (40 mg, 5.2 μmol) or TBA-**SiW10** (40 mg, 12 μmol) were quickly added to the suspension. This mixture was stirred for 10 min at room temperature, and then, acetone was removed by rotary evaporation at room temperature to obtain TBA-**Bi4**/C or TBA-**SiW10**/C.

Preparation of Ba-Bi4/C, Mg-Bi4/C, Cs-Bi4/C, and Ba-SiW10/C

Ba-**Bi4**/C was prepared by referring to the previous literature.[7] TBA-**Bi4**/C (32 mg) was added to an acetone solution (16 mL) containing $Ba(OTf)_2$ (1.6 mg, 3.7 μmol), followed by vigorous stirring for 3 min at room temperature. The black precipitates were collected by membrane filtration, washed with acetone (16 mL) three times, and dried under suction to obtain Ba-**Bi4**/C.

Mg-**Bi4**/C and Cs-**Bi4**/C were prepared by a similar procedure to Ba-**Bi4**/C, using $Mg(OTf)_2$ (1.2 mg, 3.7 μmol) and CsOTf (2.1 mg, 7.3 μmol), respectively. Ba-**SiW10**/C was prepared by a similar procedure to Ba-**Bi4**/C, using TBA-**SiW10**/C (32 mg) and $Ba(OTf)_2$ (2.0 mg, 4.7 μmol).

Preparation and characterization of Ba-Bi4

TBA-**Bi4** (100 mg, 13.1 μmol) was mixed with an acetone solution (200 mL) containing $Ba(OTf)_2$ (20.0 mg, 45.9 μmol), followed by vigorous stirring for 3 min. The precipitates were collected by membrane filtration, washed with an acetone (200 mL) three times, and dried under suction. Finally, the precipitates were dried in vacuo to obtain Ba-**Bi4**. IR (KBr pellet, cm^{-1}): 1618, 991, 944, 867, 812, 742, 531, 365, 359, 332, 320 (Figure S1).

Preparation of the gas-diffusion electrodes

The catalyst ink was prepared by ultrasonicating the mixture of the catalyst (TBA-**Bi4**/C: 17.5 mg, Ba-**Bi4**/C: 20.0 mg, Mg-**Bi4**/C: 20.0 mg, Cs-**Bi4**/C: 19.9 mg), neutralized Nafion solution (200 μL, this solution was prepared by adding 10% TBAOH-methanol solution to 5% Nafion dispersion until the pH reached 7), and acetonitrile (4 mL) for 1 h. For the preparation of the catalyst ink containing $Bi(OAc)_3$, the mixture of $Bi(OAc)_3$ (0.83 mg, 2.1 μmol), a carbon black support (15 mg), neutralized Nafion solution (200 μL), and acetonitrile (4 mL) was ultrasonicated for 1 h. For the preparation of the catalyst ink containing Ba-**SiW10**/C and $Bi(OAc)_3$ (Ba-**SiW10**/C+$Bi(OAc)_3$), the mixture of Ba-**SiW10**/C (19.5 mg), $Bi(OAc)_3$ (0.83 mg, 2.1 μmol), neutralized Nafion solution (200 μL), and acetonitrile (4 mL) was ultrasonicated for 1 h. For the preparation of the catalyst ink containing monolayer Bi_2WO_6 or bulk Bi_2WO_6, the mixture of monolayer Bi_2WO_6 or bulk Bi_2WO_6 (0.78 mg), a carbon black support (15 mg), neutralized Nafion solution (200 μL), and acetonitrile (4 mL) was ultrasonicated for 1 h. For the preparation of the catalyst ink containing a carbon black support, the mixture of a carbon black support (15 mg), neutralized Nafion solution (200 μL), and acetonitrile (4 mL) was ultrasonicated for 1 h. The catalyst ink (800 μL) was drop-cast onto a Sigracet 39 BB (2.5×2.5 cm^2) and the catalyst-loaded electrode was dried at 313 K for 3 h.

Electrochemical measurements

Constant potential electrolysis was carried out in a gas-fed electrolyzer with three-electrode system (Figure S2). This gas-fed electrolyzer is composed of the gas chamber for gas delivery and gaseous CO_2RR products collection, the catholyte chamber for catholyte circulation and liquid CO_2RR products collection, and the anolyte chamber for anolyte circulation. The gas chamber and the catholyte chamber were separated by the working electrode (WE, the catalyst-loaded electrode with catalytic area of 1.4×1.4 cm^2) while the catholyte chamber and the anolyte chamber were separated by pre-activated CEM. The reference electrode (RE, Ag/AgCl electrode filled with 3 M NaCl aqueous solution, BAS Inc.) and the counter electrode (CE, Pt mesh electrode) were placed in the catholyte chamber and the anolyte chamber, respectively. All potentials were recorded against reversible hydrogen electrode (RHE) using the following equation:

$$E_{\mathrm{RHE}} = E_{\mathrm{Ag/AgCl}} + 0.0591 \times \mathrm{pH} + 0.209 - IR$$

where pH is the pH of the catholyte after the reaction measured by HM-30G or HM-41X (DKK-TOA CORPORATION), I [A] is the current, and R [Ω] is the solution resistance determined by potentiostatic electrochemical impedance spectroscopy (EIS). The WE, RE, and CE were connected to the VMP3 or VSP-300 multichannel potentiostats (BioLogic) to perform electrochemical measurements. 2 M $KHCO_3$ aqueous solution (50 mL for the catholyte and 30 mL for the anolyte) was used as the catholyte and the anolyte, and the catholyte and the anolyte were circulated at 20 mL min^{-1} and 8 mL min^{-1}, respectively, by using a peristaltic pump (TOKYO RIKAKIKAI CO., LTD.). For constant potential electrolysis at −0.87 V_{RHE}, the volume of the catholyte was increased to 200 mL. For 9 h CO_2RR, the volume of the catholyte was increased to 250 mL. The inlet gas flow rate was controlled at 80 mL min^{-1} by 8700MC mass flow controller (KOFLOC Corp.) for CO_2 and 8500MC mass flow controller (KOFLOC Corp.) for Ar. The outlet gas flow rate was measured for each measurement by GFM-2000 flow meter (Shimadzu GLC Ltd.) to quantify gaseous products. Every experiment was conducted under ambient pressure (1 atm) and temperature (298 K). One experiment was performed for each condition.

Cyclic voltammetry was performed by using the abovementioned gas-fed electrolyzer (Figure S2). Prior to the measurement, CO_2RR was performed at −0.8 V_{RHE} for 1 h in 2 M $KHCO_3$ aqueous solution. Then, the catholyte was exchanged to fresh Ar-purged 2 M $KHCO_3$ aqueous solution and circulated at 20 mL min^{-1}. Meanwhile, Ar was introduced into the gas chamber. Finally, the circulation of the catholyte was stopped and the potential was linearly swept between −0.7 V_{RHE} and 1.0 V_{RHE} at a scan rate of 10 mV s^{-1}.

Potentiostatic EIS shown in Figure S14 was conducted by using the abovementioned gas-fed electrolyzer (Figure S2). Prior to the measurement, CO_2RR was performed at −0.8 V_{RHE} for 1 h in 2 M $KHCO_3$ aqueous solution. Subsequently, EIS measurement was carried out at −0.8 V_{RHE} with a sinus amplitude of 10 mV in a frequency range from 100 kHz to 100 mHz.

Gaseous CO_2RR products analysis

The gaseous CO_2RR products, carbon monoxide (CO) and hydrogen (H_2), were quantified by using Nexis GC-2030 gas chromatography (Shimadzu Corporation) equipped with a barrier ionization discharge (BID) detector and a ShinCarbon ST Micropacked column (2.0 m × 1.0 mm I.D., Shinwa Chemical Industries Ltd.). The carrier gas was helium (>99.99995%, TOMOE SHOKAI Co., LTD). The external standard method was used to quantify the gaseous CO_2RR products.

The Faradaic efficiency of product i (FE_i) was calculated according to the equation below:

$$\mathrm{FE}_i = \frac{n_i F C_i v P}{RTI}$$

where n_i is the number of electrons used to produce one molecule of i, F is the Faraday constant (96485 C mol^{-1}), C_i [%] is the concentration of i in the outlet gas, v [m^3 s^{-1}] is the outlet gas flow rate, P is the pressure (1.013×10^5 Pa), R is the universal gas constant (8.314 Pa·m^3 mol^{-1} K^{-1}), T is the temperature (298 K), and I [A] is the current.

Liquid CO_2RR products analysis

The liquid CO_2RR product, formate ($HCOO^-$), was quantified by referring to the previous literature.[8] The catholyte after the reaction (400 μL) was first mixed with D_2O (200 μL) and the internal standard solution (50 μL) containing 20 mM phenol and 10 mM dimethyl sulfoxide. The ^{1}H NMR measurement of this mixture was performed at 303 K using a water suppression method. The concentration of product i in the catholyte (C_i) was determined by using a 5-point calibration curve.

The Faradaic efficiency of i (FE_i) was calculated according to the equation below:

$$\mathrm{FE}_i = \frac{n_i F C_i V}{Q} \times 100$$

where n_i is the number of electrons used to produce one molecule of i, F is the Faraday constant (96485 C mol^{-1}), C_i [mol L^{-1}] is the concentration of i in the catholyte, V [L] is the volume of the catholyte (0.05 L, 0.2 L, or 0.25 L), and Q [C] is the total charge passed through the WE.

X-ray absorption fine structure (XAFS) analysis

W L_3- and Bi L_3-edges X-ray absorption fine structure (XAFS) measurements were performed at beamline BL36XU at SPring-8. The incident X-ray beam was monochromatized by using Si(111) double-crystal monochromator. The XAFS measurements under fluorescence mode were carried out using the CITIUS detector.[9] The measurements were carried out at room temperature. X-ray absorption near edge structure (XANES) and extended X-ray absorption fine structure (EXAFS) spectra were analyzed using the Athena software (Demeter, ver. 0.9.26; Bruce Ravel). XANES spectra were obtained after normalization. The k-space EXAFS spectra were obtained as k^3-weighted χ spectra after normalization. The R-space EXAFS spectra were obtained after the Fourier-transformation in the k range of 3–11 Å^{-1} and 3–13 Å^{-1} for Bi L_3-edge and W L_3-edge, respectively.

2. Supplementary Figures

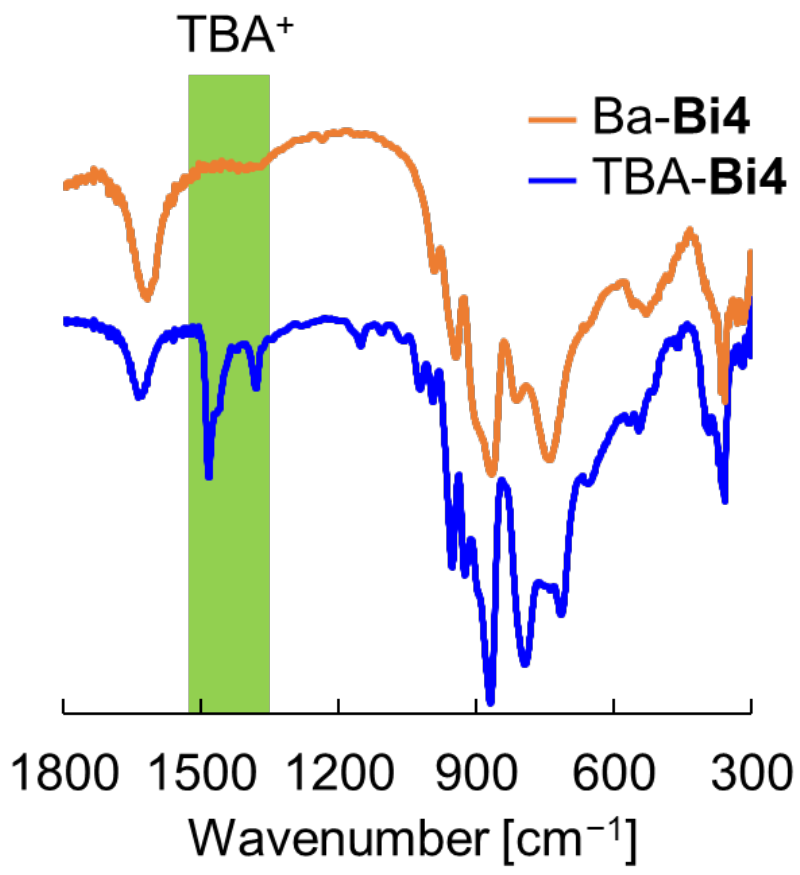

Figure S1. IR spectra of TBA-**Bi4** and Ba-**Bi4**.

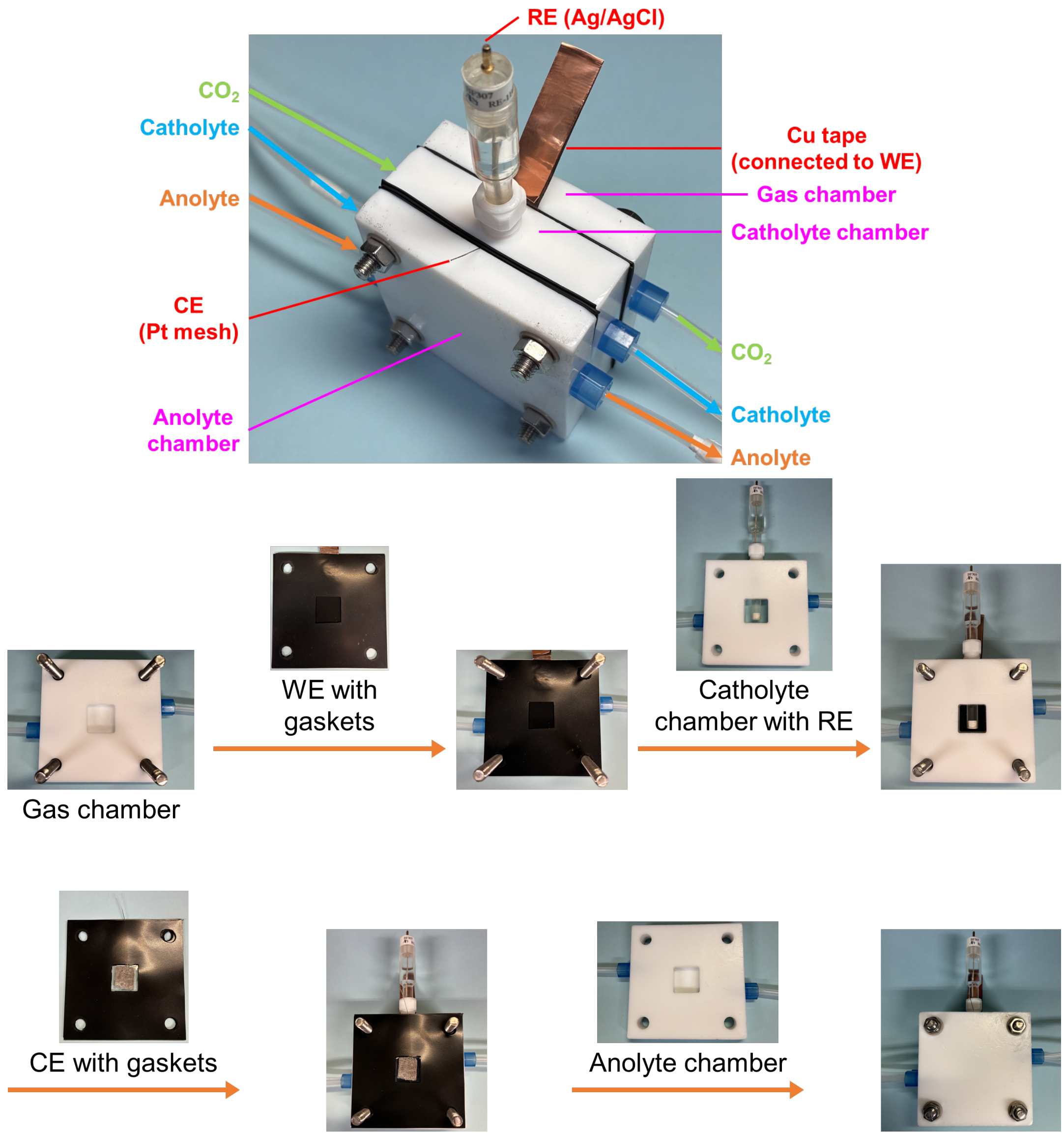

Figure S2. The configuration and assembly of a gas-fed CO_2 electrolyzer.

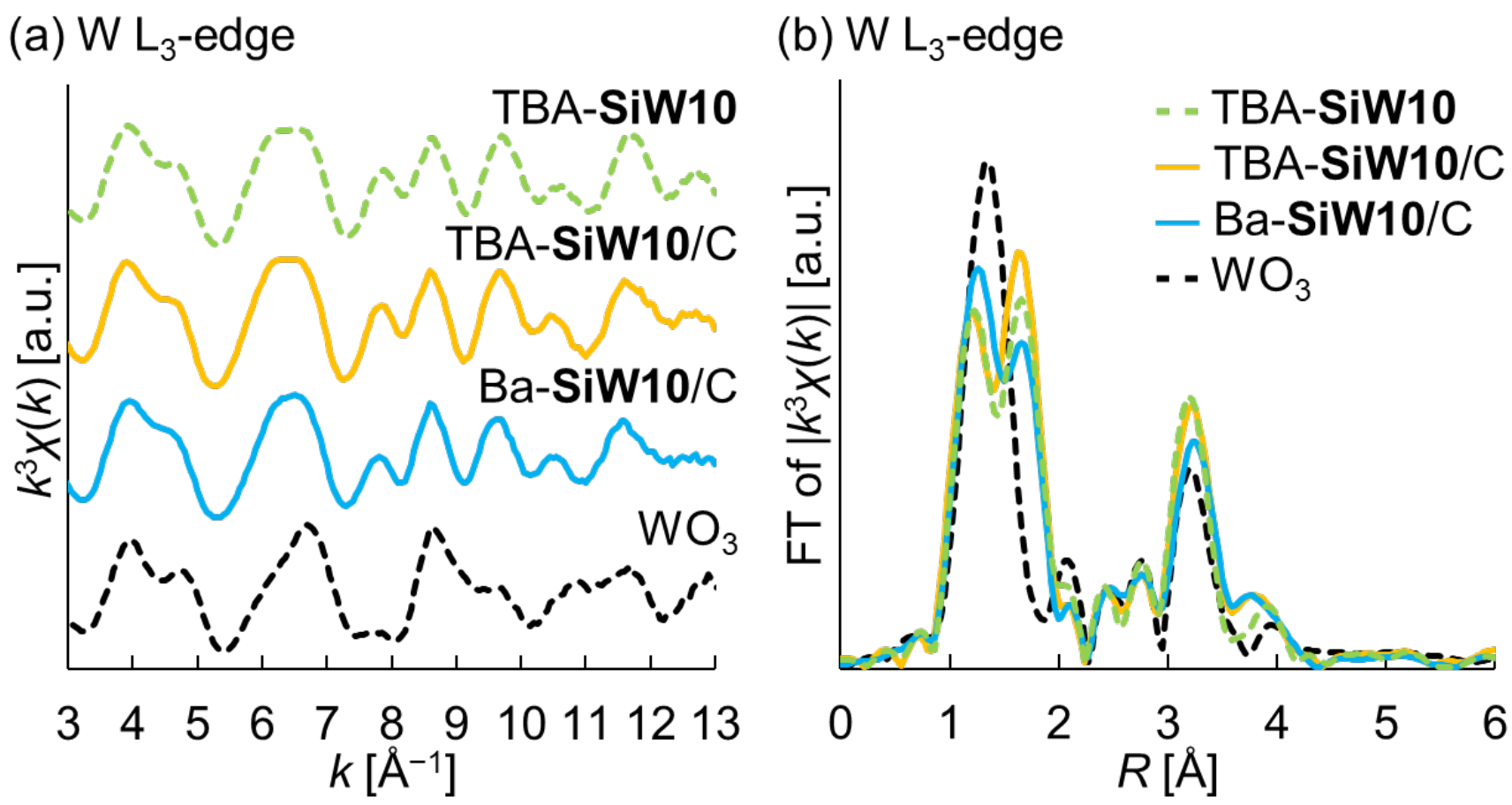

Figure S3. XAFS measurements of Ba-**SiW10**/C. (a) k^3-weighted W L_3-edge EXAFS oscillation. (b) Fourier-transformed W L_3-edge EXAFS spectra (k = 3–13 Å^{-1}).

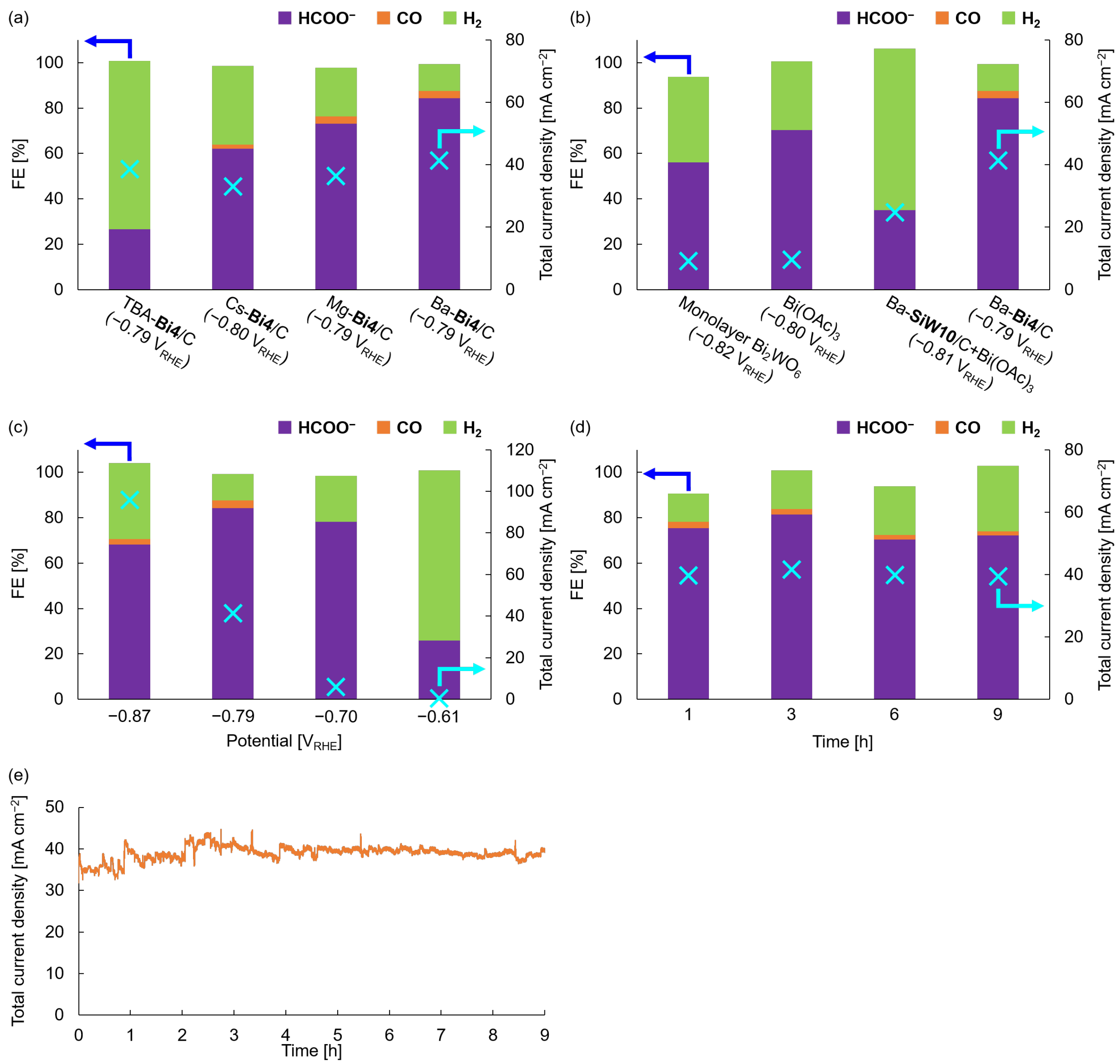

Figure S4. The CO_2RR performance of POM-based catalyst-loaded electrodes with 2 M $KHCO_3$ aqueous solution. (a) **Bi4**/C possessing various countercations at a constant potential around −0.8 V_{RHE} for 1 h. (b) Ba-**Bi4**/C and other benchmark catalysts at a constant potential around −0.8 V_{RHE} for 1 h. (c) Ba-**Bi4**/C at different potentials for 1 h. (d) Ba-**Bi4**/C at −0.82 V_{RHE} for 9 h. (e) Chronoamperometric curve of Ba-**Bi4**/C at −0.82 V_{RHE} for 9 h. All potentials were *iR*-corrected.

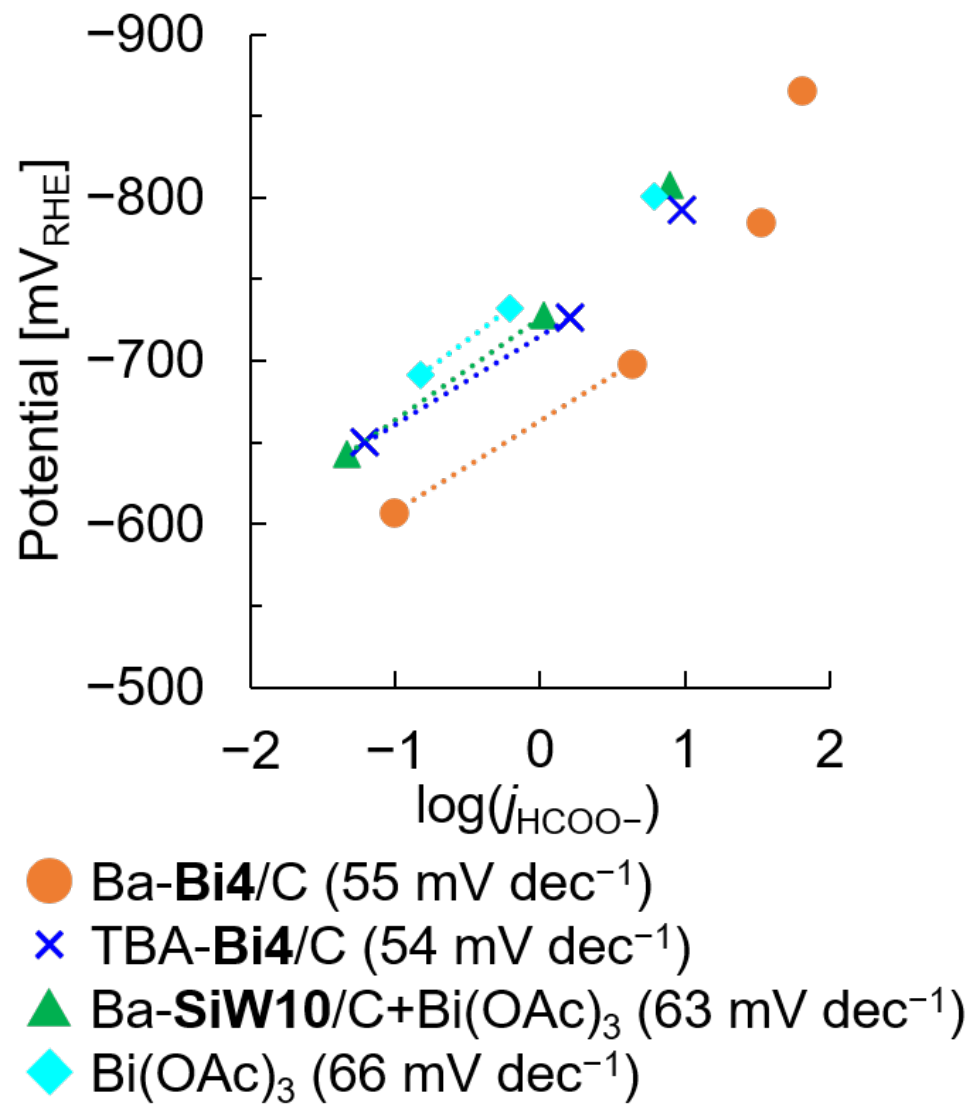

Figure S5. Tafel plots for $HCOO^-$ production over various catalyst-loaded electrodes.

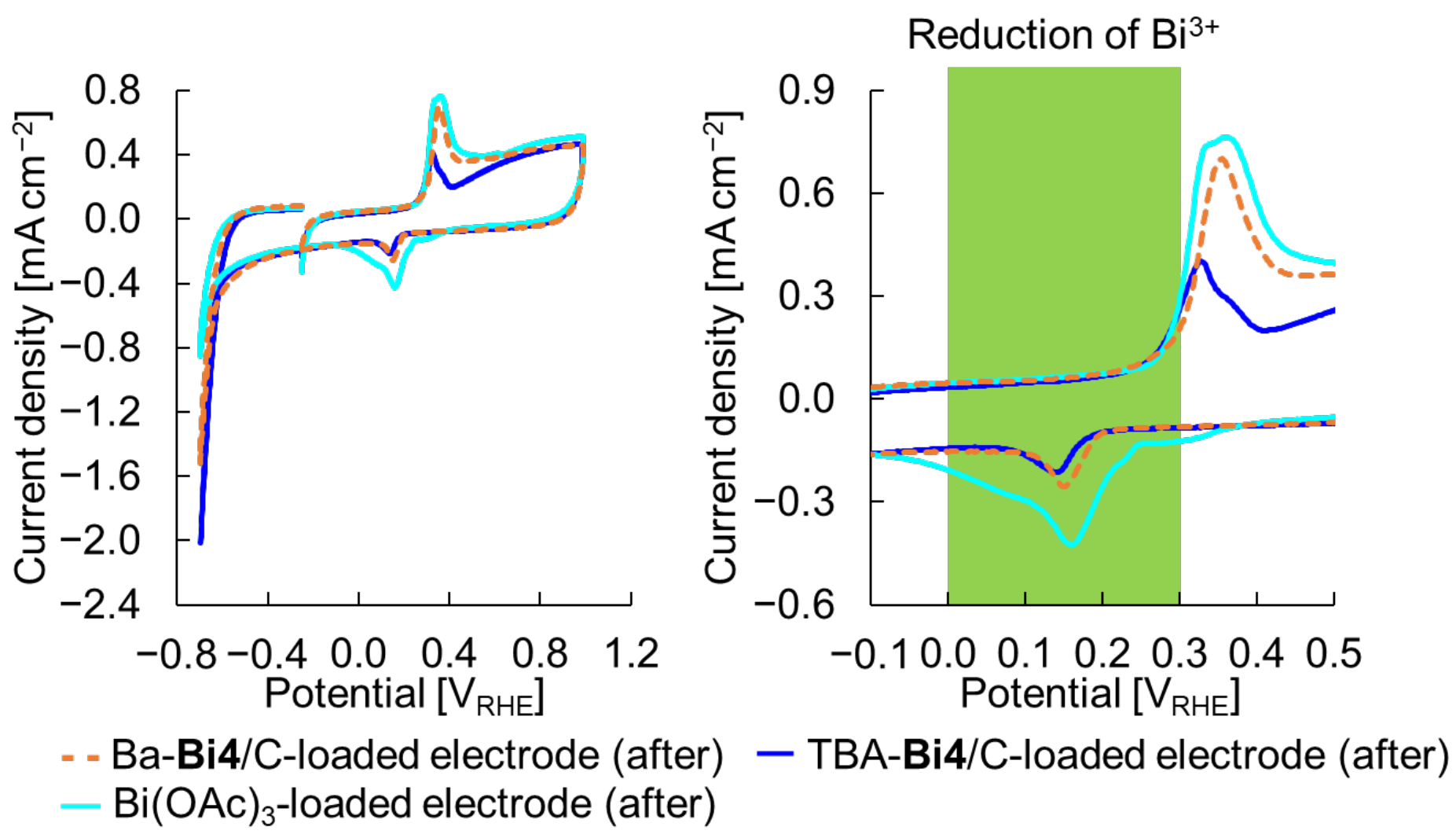

Figure S6. Cyclic voltammograms of Ba-**Bi4**/C-, TBA-**Bi4**/C-, and Bi(OAc)$_3$-loaded electrodes after a 1 h CO_2RR at −0.8 V_{RHE} (scan rate, 10 mV s^{-1}). The region corresponding to the reduction of Bi^{3+} is shown in light green.

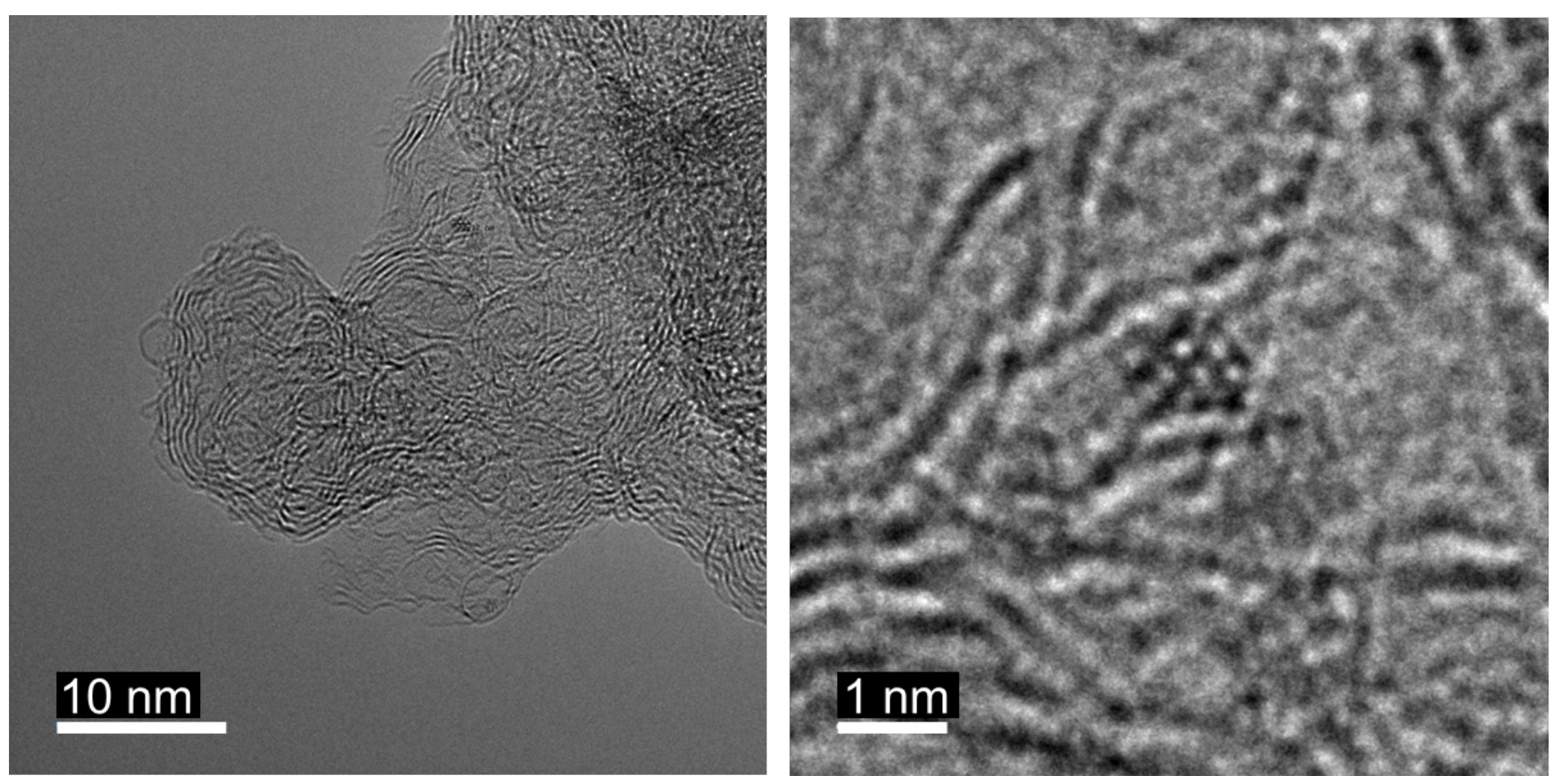

Figure S7. TEM images of Ba-**Bi4**/C-loaded electrode after 1 h of CO_2RR.

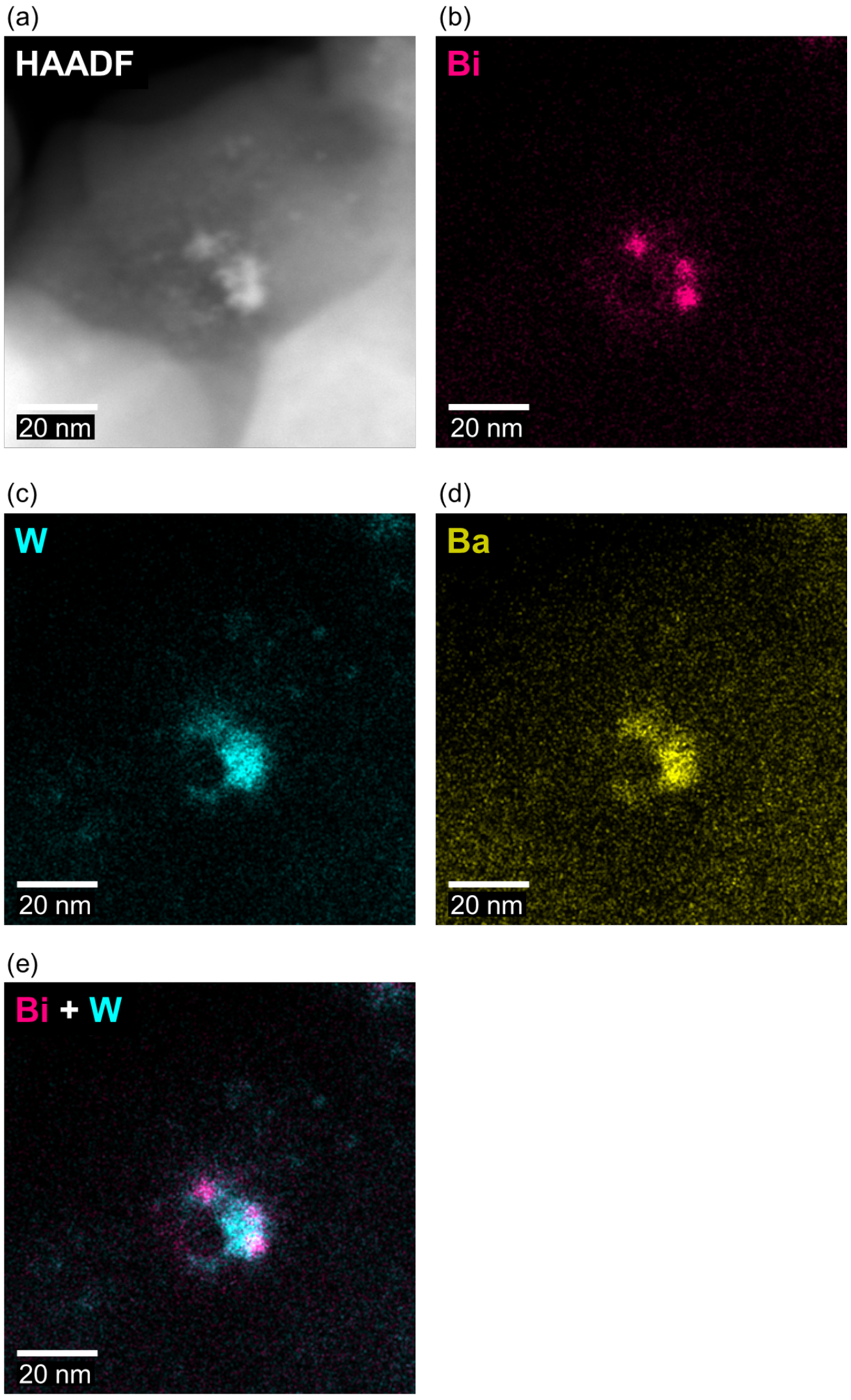

Figure S8. STEM images of Ba-**Bi4**/C-loaded electrode after 1 h of CO_2RR. (a) HAADF-STEM image and (b–e) STEM-EDS mappings (Bi, magenta; W, cyan; Ba, yellow).

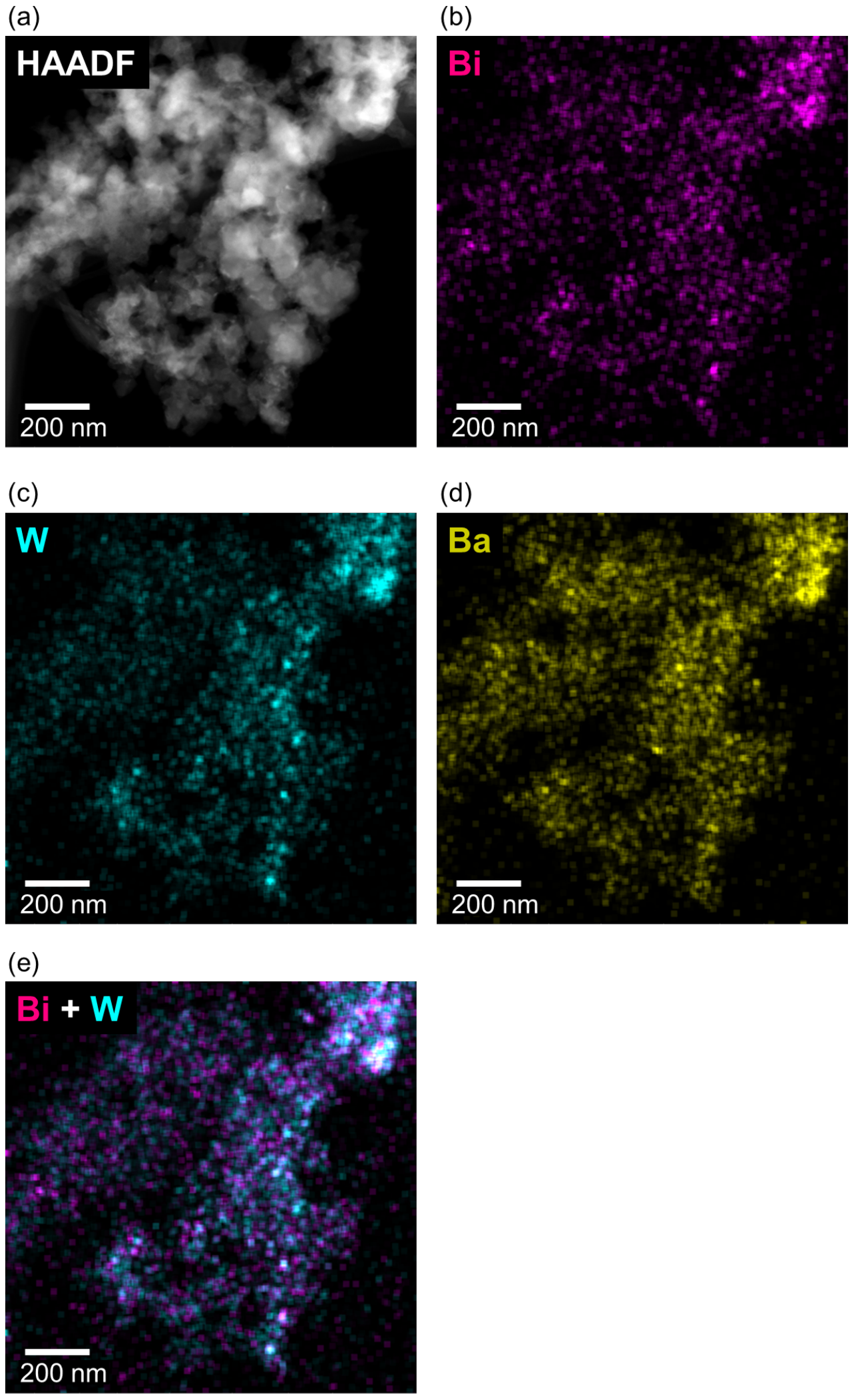

Figure S9. Low-magnification STEM images of Ba-**Bi4**/C-loaded electrode after 1 h of CO_2RR. (a) HAADF-STEM image and (b–e) STEM-EDS mappings (Bi, magenta; W, cyan; Ba, yellow).

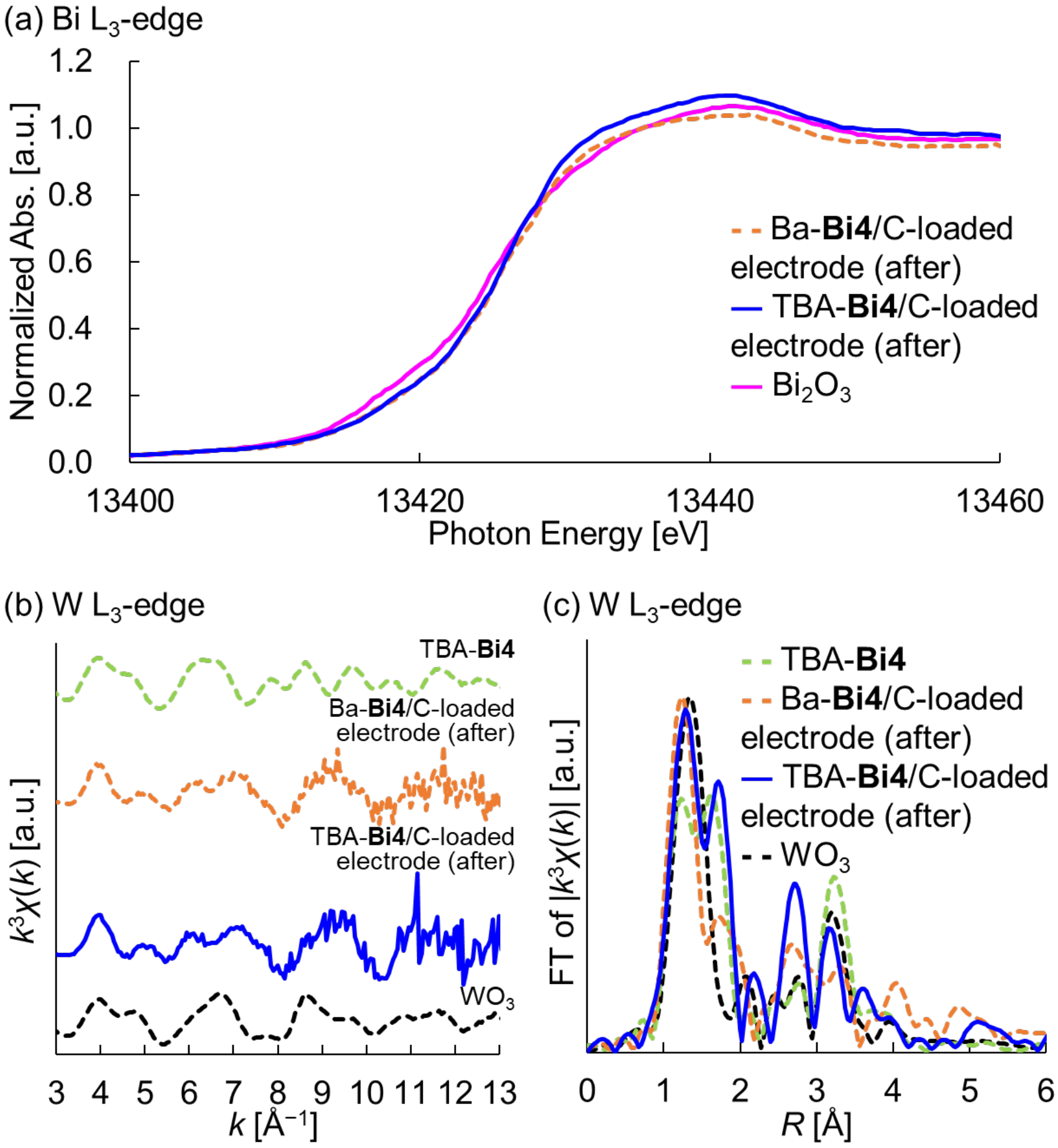

Figure S10. XAFS measurements of Ba-**Bi4**/C- and TBA-**Bi4**/C-loaded electrodes after a 1 h CO_2RR. (a) Bi L_3-edge XANES spectra. (b) k^3-weighted W L_3-edge EXAFS oscillation. (c) Fourier-transformed W L_3-edge EXAFS spectra (k = 3–13 Å^{-1}).

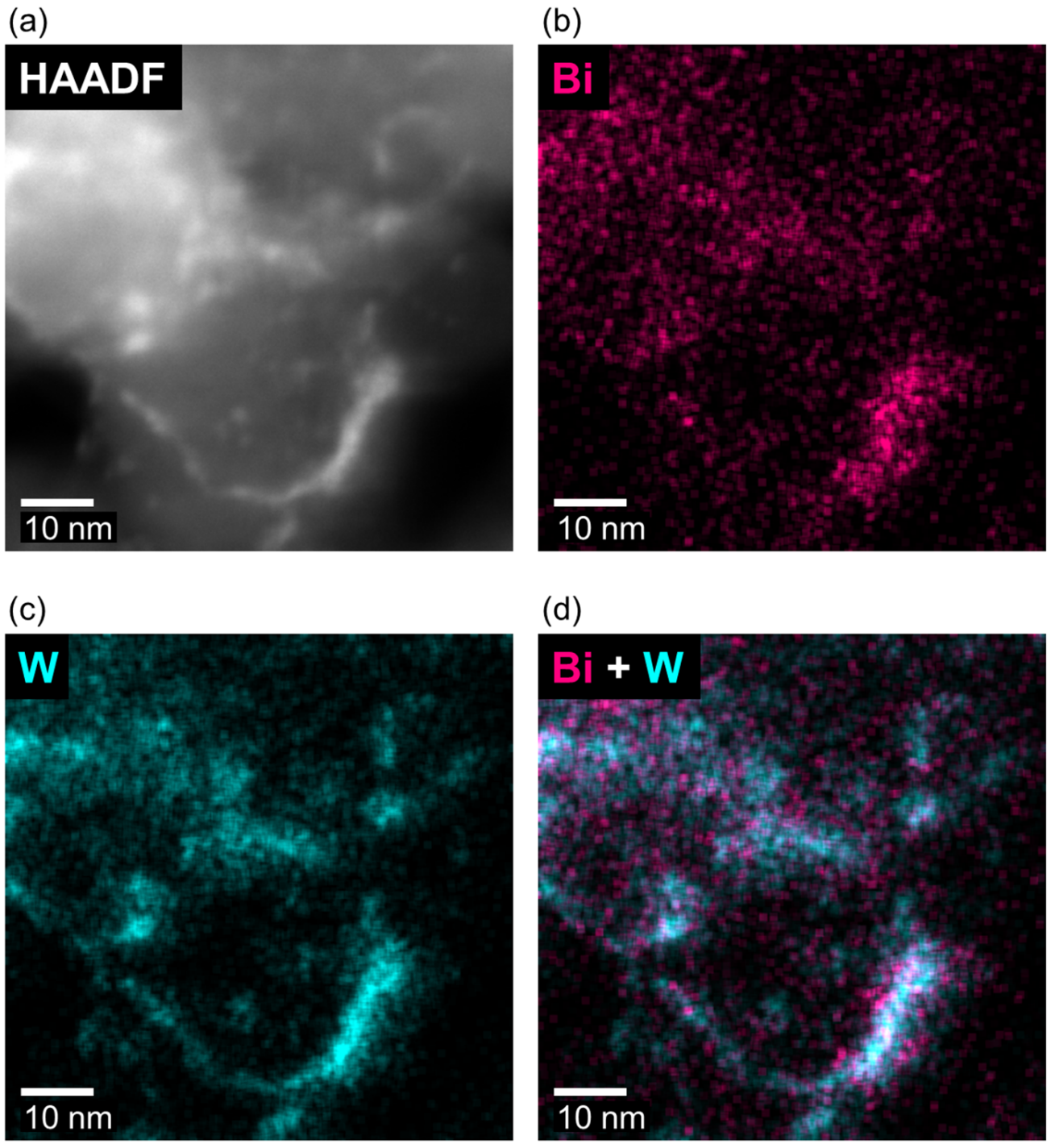

Figure S11. STEM images of TBA-**Bi4**/C-loaded electrode after a 1 h CO_2RR. (a) HAADF-STEM image and (b–d) STEM-EDS mappings (Bi, magenta; W, cyan).

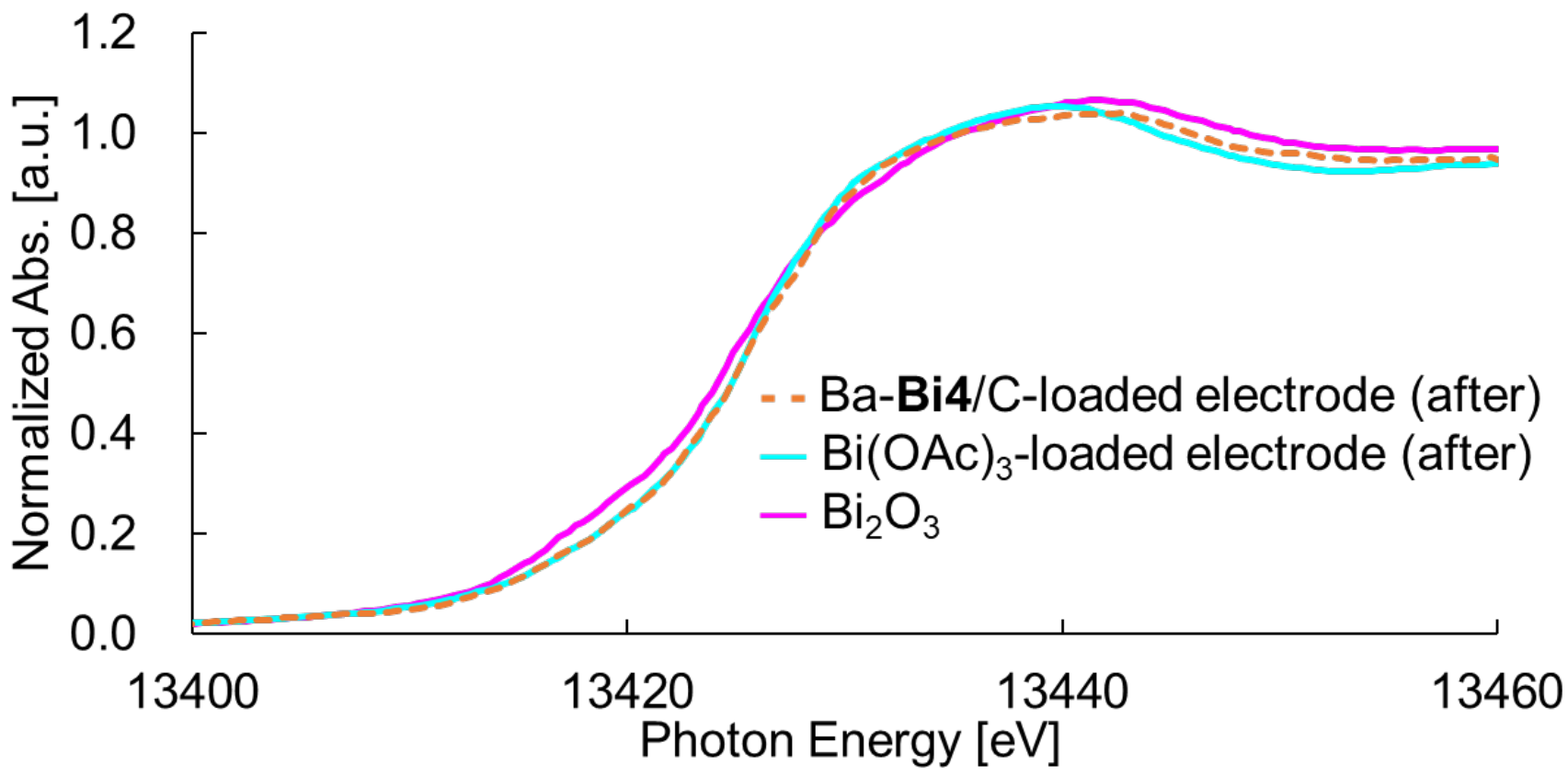

Figure S12. Bi L_3-edge XANES spectra of Ba-**Bi4**/C- and $Bi(OAc)_3$-loaded electrodes after a 1 h CO_2RR.

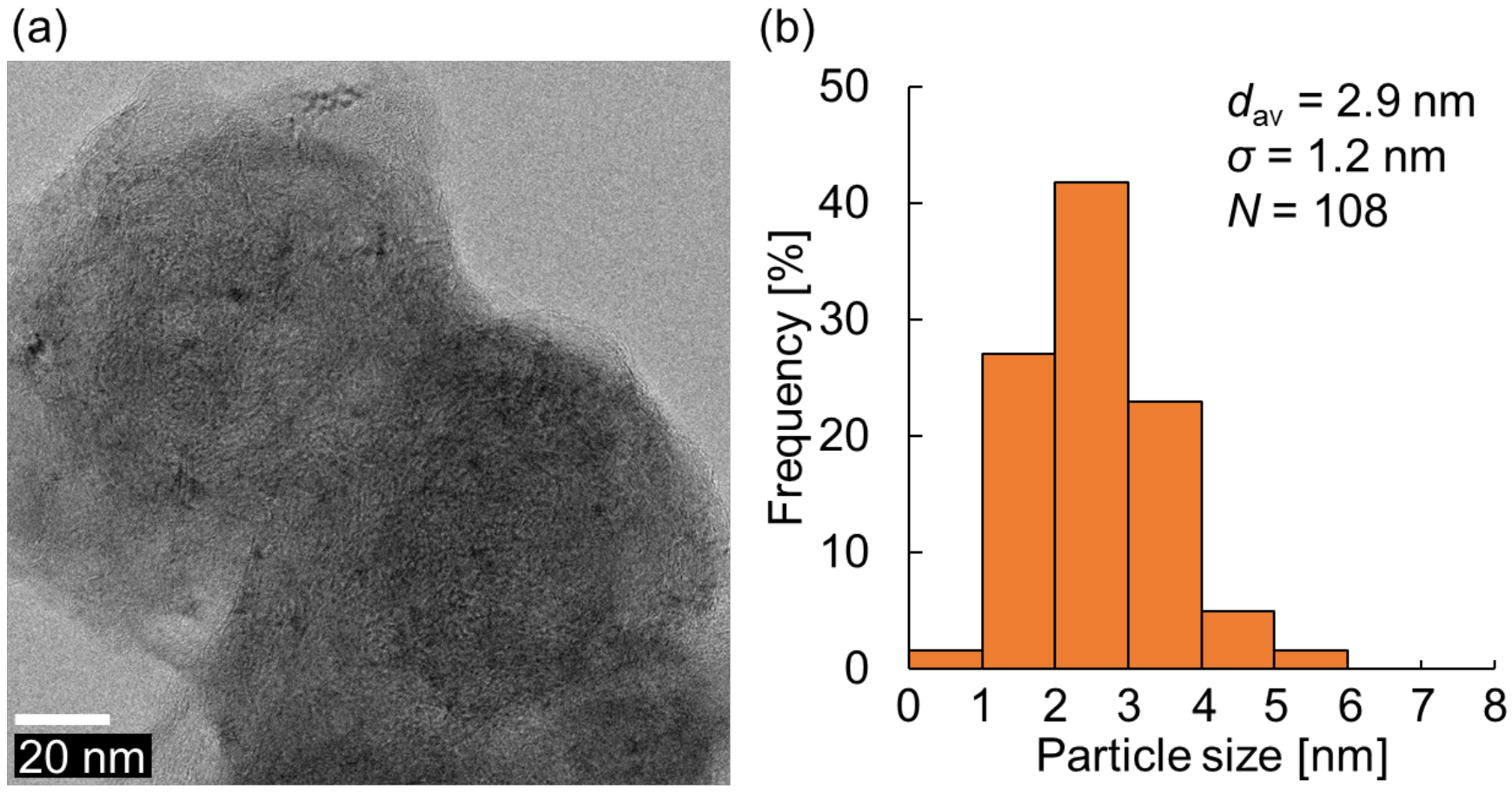

Figure S13. TEM image of $Bi(OAc)_3$-loaded electrode after a 1 h CO_2RR. (a) TEM image and (b) the corresponding size-distribution histogram.

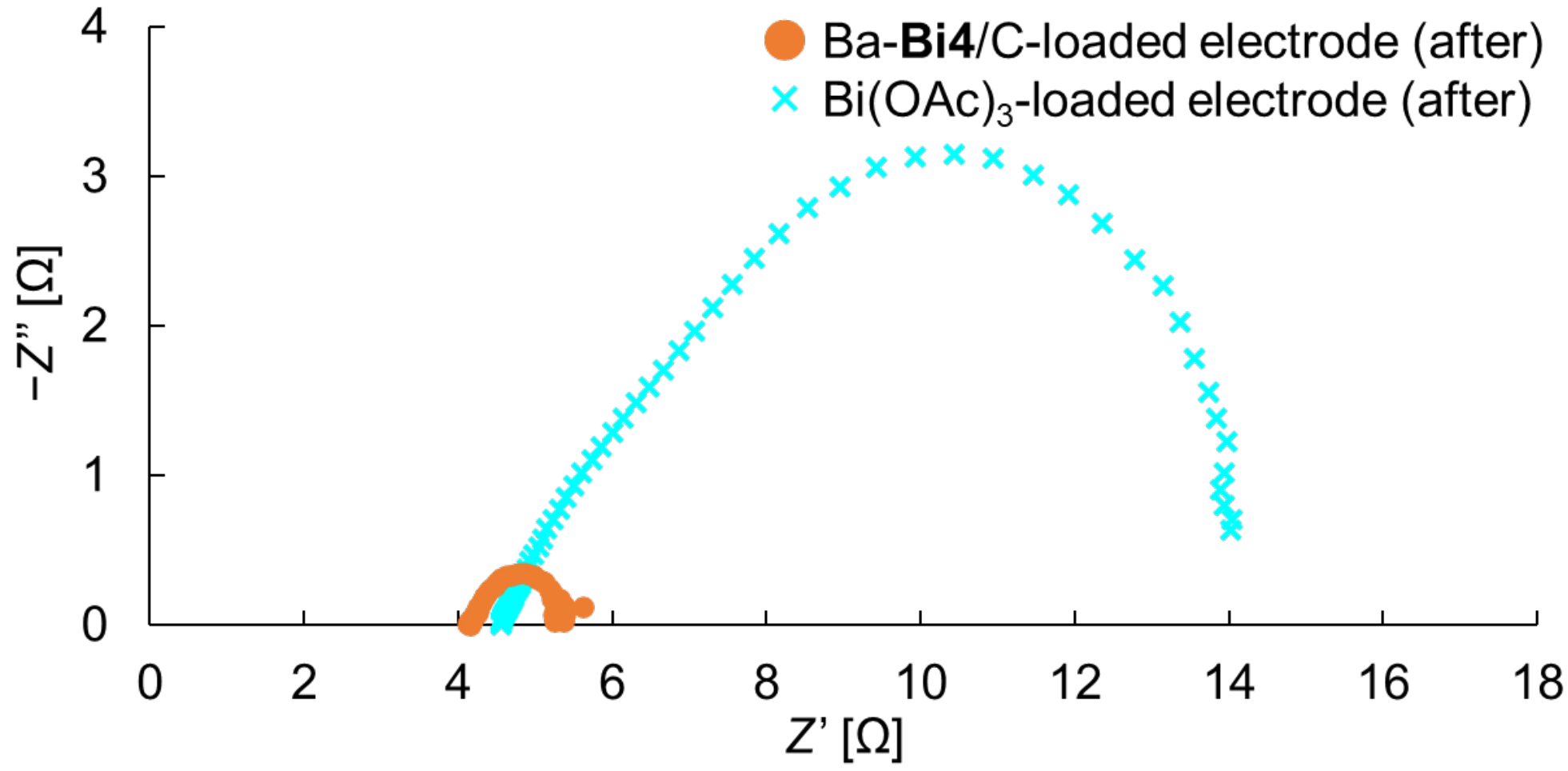

Figure S14. Nyquist plots of Ba-**Bi4**/C- and $Bi(OAc)_3$-loaded electrodes after a 1 h CO_2RR. The EIS measurements were carried out at −0.8 V_{RHE} with a sinus amplitude of 10 mV in a frequency range from 100 kHz to 100 mHz.

3. Supplementary Tables

Table S1. The summary of POM-based CO_2RR catalysts.

Catalyst	Electrolyte	FE [%]	Partial current density [mA cm^{-2}]	Reference
Ba-**Bi4**/C	2 M $KHCO_3$	84.3 ($HCOO^-$) @−0.79 V_{RHE}	33.5 ($HCOO^-$) @−0.79 V_{RHE}	This work
$[MTRP]^{n+}/[SiW_{12}O_{40}]^{4-}$ (M = Mn^{3+}, Zn^{2+}, Ni^{2+})[a]	0.1 M $NaClO_4$	-	-	[10]
$(TOA)_6$[α-$SiW_{11}O_{39}$Co(_)][b]	0.1 M $TBABF_4$-CH_2Cl_2 containing 40 mM H_2O and 10 mM $TBAHSO_4$	40 (HCHO) @−1.5 $V_{Hg2Cl2/Hg}$	-	[11]
AgNC@BSA–SiW[c]	0.1 M $TBAPF_6$-*N,N*-dimethlyformamide containing 1% H_2O	75.6 (CO) @−2.32 $V_{Fc/Fc+}$	-	[12]
Ag–PMo nanocomposites[d]	0.1 M $TBAPF_6$-*N,N*-dimethlyformamide containing 0.5% H_2O	*ca*. 90 (CO) @−1.9–−2.5 $V_{Fc/Fc+}$	-	[13]
Co-PMOF[e]	0.5 M $KHCO_3$	98.7 (CO) @−0.8 V_{RHE}	*ca*. 29 (CO) @−1.0 V_{RHE}	[14]
Bi-PMo nanosheets	0.5 M $NaHCO_3$	93 ± 2 ($HCOO^-$) @−0.86 V_{RHE}	*ca*. 39 ($HCOO^-$) @−1.16 V_{RHE}	[15]
$TBA_3[H_2PW_{11}O_{39}\{Rh^{III}Cp^*(OH_2)\}]$	0.1 M $TBABF_4$-acetonitrile containing 5% H_2O	4.5 ($HCOO^-$) @−1.6 $V_{Ag/Ag+}$	-	[16]
SiW_{12}-MnL/KB[f]	0.5 M $KHCO_3$	95 (CO) @−0.72 V_{RHE}	*ca*. 14 (CO) @−0.72 V_{RHE}	[17]
PTC-255[g]	0.1 M $KHCO_3$	29.4 (CO) @−0.8 V_{RHE}	-	[18]
Indium sheet	0.1 M Na_2SO_4 containing 2 mM SiW_9V_3	96.5 (acetate) @−0.71 V_{RHE}<br>56.82 ($HCOO^-$) @−1.6 $V_{Ag/AgCl}$	*ca*. 0.6 (acetate) @−0.71 V_{RHE}<br>*ca*. 2.6 ($HCOO^-$) @−1.7 $V_{Ag/AgCl}$	[19]

a TRP = μ-(meso-5,10,15,20-tetra(pirydil)porphyrin)tetrakis{bis(bipyridine)chloride ruthenium(II)}
b $TOA^+ = (n\text{-}C_8H_{17})_4N^+$
c AgNC@BSA = Bovine serum albumin-capped silver nanoclusters, SiW = $[\alpha\text{-}SiW_{12}O_{40}]^{4-}$
d PMo = $[PMo_{12}O_{40}]^{3-}$
e Co-PMOF = $[PMo^{V}_8Mo^{VI}_4O_{35}(OH)_5Zn_4]_2$[Co-TCPP]$[2H_2O]$[1.5TBAOH], TCPP = tetrakis[4-carboxyphenyl]-porphyrin
f SiW_{12}-MnL = $[Mn^I(bipy)(CO)_3(CH_3CN)]_4(SiW_{12}O_{40})\cdot 5CH_3CN$, KB = Ketjen black
g PTC-255 = $Ag_2Ti_{12}(\mu_3\text{-}O)_{12}(\mu_2\text{-}O)_2(4\text{-}FBA)_{14}(\mu_2\text{-}OMe)_4(OMe)_4\cdot 2CH_3CN$, 4-FBA = 4-fluorobenzoate

Table S1. The summary of POM-based CO_2RR catalysts (continued).

Catalyst	Electrolyte	FE [%]	Partial current density [mA cm^{-2}]	Reference
Indium sheet	0.1 M Na_2SO_4 containing 2 mM $SiW_{11}Mn$	72.1 (acetate) @−1.0 $V_{Ag/AgCl}$<br>33 ($HCOO^-$) @−1.6 $V_{Ag/AgCl}$	*ca*. 0.4 (acetate) @−1.0 $V_{Ag/AgCl}$<br>*ca*. 1.5 ($HCOO^-$) @−1.6 $V_{Ag/AgCl}$	[20]
Cu_9S_5 single-unit-cell nanowires[h]	0.1 M $KHCO_3$	82.0 ($HCOO^-$) @−0.8 V_{RHE}	3.20 ($HCOO^-$) @−0.9 V_{RHE}	[21]
Mo_8@Cu/titanium dioxide nanotube arrays	saturated $NaHCO_3$	48.68 (acetate) @−1.13 V_{RHE}	*ca*. 69 (acetate) @−1.33 V_{RHE}	[22]
Zn–CoTAPc/PMo12 molecular layer sandwich nanosheets[i]	1 M $KHCO_3$	97.2 (CO) @−0.8 V_{RHE}<br>*ca*. 82 (CO) @−0.95 V_{RHE}	108 (CO) @−0.8 V_{RHE}<br>*ca*. 115 (CO) @−0.95 V_{RHE}	[23]
$\{[Cu^{II}_3(tybm)_3(H_2O)_4][\alpha\text{-A-}TeMo_6O_{24}]\}_2\cdot(\alpha\text{-A-}H_6TeMo_6O_{24})\cdot16H_2O$[j]	0.5 M $KHCO_3$	93.4 (CO) @−0.8 V_{RHE}	-	[24]
$[Ag_{11}Na_3(L_1)_4(L_2)_2(H_2O)_4(H_2PMo_9^{VI}Mo_3^{V}O_{40})_2]$[k]	$KHCO_3$<br>(concentration not reported)	97 (CO) @−0.8 V_{RHE}	-	[25]
$[Zn(MET)_2(H_2O)_2][H_2(\gamma\text{-}Mo_8O_{26})]$[l]	0.5 M $KHCO_3$	92.8 (CO) @−0.8 V_{RHE}	-	[26]
Au–PW_{12} sub-1 nm nanowires	0.1 M $KHCO_3$	90.64 (CO) @−0.9 V_{RHE}	-	[27]
H-POM@PCN-222(Co)[m]	0.5 M $KHCO_3$	96.2 (CO) @−0.8 V_{RHE}	16.56 (CO) @−1.0 V_{RHE}	[28]
$\{SiCu^{II}_3W_9\}$[n]	0.1 M $TBAPF_6$-acetonitrile	98 (CO) @−2.5 $V_{Fc/Fc+}$	-	[29]

h nanowires composed of Cu_9S_5 unit cell and **PW12**
i CoTAPc = cobalt tetraaminophthalocyanine
j tybm = 4-[1,3,4]triazol-4-yl-benzylamine
k L_1 = 1H-1,2,3,5,10-pentaaza-cylopenta[b]anthracene-4,11-dione, L_2 = 6-nitro-1H-benzotriazole
l MET = 4-(3-imidazol-1-yl-ethyl)-4*H*-[1,3,4]triazole
m POM = $Co^{III}Co^{II}(H_2O)W_{11}O_{39}$
n $\{SiCu^{II}_3W_9\}$ = $\{Si[Cu(H_2O)]_3W_9O_{37}\}^{10-}$

Table S1. The summary of POM-based CO_2RR catalysts (continued).

Catalyst	Electrolyte	FE [%]	Partial current density [mA cm^{-2}]	Reference
Indium sheet	acetonitrile containing 2 mM SVW_{11}	67.4 (acetic acid) @−0.5 $V_{Ag/Ag+}$	-	[30]
Co-POMOF^{o}	0.5 M $KHCO_3$	>99 (CO) @−1.0 V_{RHE}	*ca.* 22 (CO) @−1.2 V_{RHE}	[31]
**{$Ag_{49}Mo_{16}$}**^{p}	0.5 M $KHCO_3$	44.75 (CO) @−0.8 V_{RHE}	*ca.* 8 (CO) @−1.0 V_{RHE}	[32]
$TBA_3H[(Ru(dmbpy)(ppt)Cl)_2P_2W_{17}O_{57}]^{q}$	0.1 M $TBAPF_6$-acetonitrile containing acetic acid	67 (formic acid) @−1.56 V_{NHE}	-	[33]
$P_2W_{18}Mn_4$@PCN-222	0.5 M $KHCO_3$	72.6 (CO) @−0.60 V_{RHE}	*ca.* 0.4 (CO) @−0.65 V_{RHE}	[34]
Indium sheet	acetonitrile containing 2 mM $SVMo_{11}$	69.5 (ethanol) @−1.1 $V_{Ag/Ag+}$	*ca.* 0.1 (ethanol) @−1.1 $V_{Ag/Ag+}$	[35]
$Cu_4[\alpha_2\text{-}P_2W_{17}O_{61}In(OH)]$-decorated indium hydroxide nanocrystals	0.1 M $KHCO_3$	*ca.* 10 (CO) @−0.6 V_{RHE}	-	[36]
Indium sheet	0.1 M Na_2SO_4 containing 2 mM PV_3Mo_9	93.4 (ethanol) @−0.3 V_{RHE}	-	[37]
$CuInSe_2$	0.1 M Na_2SO_4 containing 2 mM PV_3Mo_9	68.0 (CO) @−0.1 V_{RHE}	29.3 (CO) @−0.6 V_{RHE}	[38]
Ba-**Pd2**/C^{r}	1 M $KHCO_3$	92.9 ± 4.8 (CO) @−0.80 V_{RHE}<br>50.9 ± 5.5 (CO) @−0.85 V_{RHE}	22.5 ± 0.6 (CO) @−0.80 V_{RHE}<br>51 ± 3 (CO) @−0.85 V_{RHE}	[39]
$P_2W_{18}Co_4$@PCN-222	0.5 M $KHCO_3$	72 (CO) @−0.75 V_{RHE}	*ca.* 3 (CO) @−0.85 V_{RHE}	[40]
H-$SiW_{11}Co$@PCN-224	0.5 M $KHCO_3$	89.9 (CO) @−0.80 V_{RHE}	*ca.* 6 (CO) @−0.90 V_{RHE}	[41]
{$SiCuFeGaW_9$}^{s}	0.1 M $TBAPF_6$-acetonitrile	96 (CO) @−2.5 $V_{Fc/Fc+}$	-	[42]

o Co-POMOF = $[PMo^{V}_8Mo^{VI}_4O_{35}(OH)_5Zn^{II}_4]_2[Co^{II}\text{-TCPP}][H_2O]$·Guest, TCPP = tetrakis(4-carboxyphenyl)-porphyrin

p **{$Ag_{49}Mo_{16}$}** = $[(Mo_6O_{22})@H_3Ag_{49}(MoO_3)_9(MoO_4)(TC4A)_6(^{i}PrS)_{18}(CH_3CN)_2(H_2O)]$, TC4A = thiacalix[4]arene

q dmbpy = 4,4'-dimethyl-2,2'-dipyridyl, ppt = 4'-(4-phosphonophenyl)-2,2':6',2''-terpyridine

r **Pd2** = $[\gamma\text{-}H_2SiW_{10}O_{36}Pd_2(OAc)_2]^{4-}$

s {$SiCuFeGaW_9$} = $[SiCu^{II}Fe^{III}Ga^{III}(H_2O)_3W_9O_{37}]^{8-}$

Table S1. The summary of POM-based CO_2RR catalysts (continued).

Catalyst	Electrolyte	FE [%]	Partial current density [mA cm^{-2}]	Reference
$H_2\{Cu_2$(6-nbm)$_4$[$TeMo_6O_{24}$](H_2O)$_4\}$·(6-nbm)$_2$·4H_2O^t	0.5 M $KHCO_3$	-	-	[43]
Indium sheet	0.1 M Na_2SO_4 containing 2 mM $PVMoW_{10}$	75.6 (acetate) @−1.0 $V_{Ag/AgCl}$	-	[44]
Indium sheet	0.1 M Na_2SO_4 containing 2 mM PV_2MoW_9	85.1 (ethanol) @−0.7 $V_{Ag/AgCl}$	-	[44]
ZnAl-W_{10}-3	0.1 M $KHCO_3$	*ca*. 67 (CO)^u	*ca*. 50 (CO)^u	[45]
CoT-7	0.5 M $KHCO_3$	98.1 (CO) @−0.9 V_{RHE}	32 (CO) @−1.0 V_{RHE}	[46]
Cu_1In_3	0.1 M Na_2SO_4 containing 2 mM PV_2MoW_9	95.3 (CO) @−0.25 V_{RHE}	64.9 (CO) @−0.55 V_{RHE}	[47]
POM-CuSe/rGO	0.1 M Na_2SO_4 containing 2 mM PV_3Mo_9	*ca*. 60 (CO) @−0.7 V_{RHE}	*ca*. 120 (CO) @−1.1 V_{RHE}	[48]
CoW_{11}Co@ZIF-8	0.25 M K_2SO_4	72.4 (CO) @−1.10 V_{RHE}	*ca*. 4.4 (CO) @−1.3 V_{RHE}	[49]
Ba-**AuAg**/C^v	1 M KOH	95.3 ± 4.2 (CO) @−0.39 V_{RHE}	171 ± 4 (CO) @−0.39 V_{RHE}	[7]
Au1Ag2-**SiW9**/C^w	1 M $KHCO_3$	81.1 (CO) @−0.69 V_{RHE}	28.2 (CO) @−0.69 V_{RHE}	[50]

t 6-nbm = 6-nitrobenzimidazole
u constant current electrolysis carried out at the total current density of 80 mA cm^{-2}
v **AuAg** = $[Au_8Ag_{26}(P_8W_{48}O_{184})]^{24-}$
w carbon-supported Au–Ag nanoparticles stabilized by **SiW9**

Table S2. The results of ICP-AES measurements of the catalysts.

Sample	wt%			Molar ratio		
	Bi	W	Ba	Bi	W	Ba
TBA-**Bi4**/C	2.56	11.53	-	3.91	20	-
Ba-**Bi4**/C	2.24	9.95	1.30	3.96	20	3.50
TBA-**SiW10**/C	-	11.85	-	-	10	-
Ba-**SiW10**/C	-	10.22	1.51	-	10	1.98

Table S3. The results of CO_2RR around −0.8 V_{RHE} for 1 h in 2 M $KHCO_3$ aqueous solution using various catalysts.

Catalyst	Potential [V_{RHE}]	Total current density [mA cm^{-2}]	Partial current density *j* [mA cm^{-2}]			Faradaic efficiency FE [%]		
			$HCOO^-$	CO	H_2	$HCOO^-$	CO	H_2
Ba-**Bi4**/C	−0.79	41.4	33.5	1.4	4.9	84.3	3.3	11.8
Mg-**Bi4**/C	−0.79	36.4	25.0	1.1	7.8	73.2	3.1	21.5
Cs-**Bi4**/C	−0.80	33.1	19.6	0.6	11.4	62.1	1.8	34.6
TBA-**Bi4**/C	−0.79	38.6	9.6	n.d.	28.6	26.6	n.d.	74.2
Ba-**SiW10**/C+Bi(OAc)$_3$	−0.81	24.7	7.8	n.d.	17.6	35.0	n.d.	71.1
Bi(OAc)$_3$	−0.80	9.5	6.1	n.d.	2.9	70.4	n.d.	30.3
Monolayer Bi_2WO_6	−0.82	9.1	4.5	n.d.	3.4	56.1	n.d.	37.7
Bulk Bi_2WO_6	−0.82	9.5	1.3	n.d.	7.1	17.1	n.d.	74.5

Table S4. The results of control experiments for 1 h in 2 M $KHCO_3$ aqueous solution using a carbon black support (C) and Ba-**Bi4**/C.

Catalyst	Atmosphere	Potential [V_{RHE}]	Total current density [mA cm^{-2}]	Partial current density *j* [mA cm^{-2}]			Faradaic efficiency FE [%]		
				$HCOO^-$	CO	H_2	$HCOO^-$	CO	H_2
C	CO_2	−0.81	2.2	n.d.	n.d.	2.3	n.d.	n.d.	104.5
Ba-**Bi4**/C	Ar	−0.79	35.4	n.d.	n.d.	34.6	n.d.	n.d.	97.6
Ba-**Bi4**/C	CO_2	−0.79	41.4	33.5	1.4	4.9	84.3	3.3	11.8

Table S5. The results of CO_2RR at different potentials for 1 h in 2 M $KHCO_3$ aqueous solution using Ba-**Bi4**/C.

Potential [V_{RHE}]	Total current density [mA cm^{-2}]	Partial current density *j* [mA cm^{-2}]			Faradaic efficiency FE [%]		
		$HCOO^-$	CO	H_2	$HCOO^-$	CO	H_2
−0.61	0.4	0.1	n.d.	0.3	25.9	n.d.	75.0
−0.70	6.0	4.3	n.d.	1.2	78.2	n.d.	20.3
−0.79	41.4	33.5	1.4	4.9	84.3	3.3	11.8
−0.87	95.8	63.9	2.2	32.2	68.3	2.3	33.6

Table S6. The results of CO_2RR at −0.82 V_{RHE} for 9 h in 2 M $KHCO_3$ aqueous solution using Ba-**Bi4**/C.

Time [h]	Total current density [mA cm^{-2}]	Partial current density *j* [mA cm^{-2}]			Faradaic efficiency FE [%]		
		$HCOO^-$	CO	H_2	$HCOO^-$	CO	H_2
1	39.7	27.2	1.1	5.0	75.5	2.7	12.6
3	41.6	32.2	1.0	7.0	81.5	2.4	16.9
6	39.8	27.9	0.8	8.5	70.4	2.1	21.4
9	39.4	28.1	0.7	11.4	72.2	1.8	29.0

Table S7. The results of ICP-AES measurements of the catalyst-loaded electrodes before and after a 1 h CO_2RR.

Sample	Before		After	
	Bi [wt%]	W [wt%]	Bi [wt%]	W [wt%]
TBA-**Bi4**/C	0.117	0.533	0.053	0.122
Ba-**Bi4**/C	0.115	0.541	0.044	0.049
$Bi(OAc)_3$	0.190	-	0.097	-

4. References

[1] K. P. Kuhl, E. R. Cave, D. N. Abram, T. F. Jaramillo, *Energy Environ. Sci.* **2012**, *5*, 7050.

[2] T. Hanaya, K. Suzuki, R. Sato, K. Yamaguchi, N. Mizuno, *Dalton Trans.* **2017**, *46*, 7384.

[3] K. Kamata, K. Yonehara, Y. Sumida, K. Yamaguchi, S. Hikichi, N. Mizuno, *Science* **2003**, *300*, 964.

[4] T. Suzuki, T. Yabe, K. Wachi, K. Yonesato, K. Suzuki, K. Yamaguchi, *ChemNanoMat* **2023**, *9*, e202200428.

[5] S. Liu, C. Wang, J. Wu, B. Tian, Y. Sun, Y. Lv, Z. Mu, Y. Sun, X. Li, F. Wang, Y. Wang, L. Tang, P. Wang, Y. Li, M. Ding, *ACS Catal.* **2021**, *11*, 12476.

[6] H. Hori, M. Takase, F. Amano, B. Ohtani, *Chem. Lett.* **2015**, *44*, 1723.

[7] K. Kawakami, K. Yonesato, S. Kikkawa, S. Yamazoe, T. Kaneko, T. Uruga, F. Amano, Y. Honjo, T. Hatsui, K. Yamaguchi, K. Suzuki, *ChemCatChem* **2026**, *18*, e01423.

[8] S. Gonglach, S. Paul, M. Haas, F. Pillwein, S. S. Sreejith, S. Barman, R. De, S. Müllegger, P. Gerschel, U.-P. Apfel, H. Coskun, A. Aljabour, P. Stadler, W. Schöfberger, S. Roy, *Nat. Commun.* **2019**, *10*, 3864.

[9] Y. Takahashi, M. Abe, H. Uematsu, S. Takazawa, Y. Sasaki, N. Ishiguro, K. Ozaki, Y. Honjo, H. Nishino, K. Kobayashi, T. N. Hiraki, Y. Joti, T. Hatsui, *J. Synchrotron Rad.* **2023**, *30*, 989.

[10] M. García, M. J. Aguirre, G. Canzi, C. P. Kubiak, M. Ohlbaum, M. Isaacs, *Electrochim. Acta* **2014**, *115*, 146.

[11] M. Girardi, S. Blanchard, S. Griveau, P. Simon, M. Fontecave, F. Bedioui, A. Proust, *Eur. J. Inorg. Chem.* **2015**, 3642.

[12] S.-X. Guo, D. R. MacFarlane, J. Zhang, *ChemSusChem* **2016**, *9*, 80.

[13] S.-X. Guo, F. Li, L. Chen, D. R. MacFarlane, J. Zhang, *ACS Appl. Mater. Interfaces* **2018**, *10*, 12690.

[14] Y.-R. Wang, Q. Huang, C.-T. He, Y. Chen, J. Liu, F.-C. Shen, Y.-Q. Lan, *Nat. Commun.* **2018**, *9*, 4466.

[15] S.-X. Guo, Y. Zhang, X. Zhang, C. D. Easton, D. R. MacFarlane, J. Zhang, *ChemSusChem* **2019**, *12*, 1091.

[16] M. Girardi, D. Platzer, S. Griveau, F. Bedioui, S. Alves, A. Proust, S. Blanchard, *Eur. J. Inorg. Chem.* **2019**, 387.

[17] J. Du, Z.-L. Lang, Y.-Y. Ma, H.-Q. Tan, B.-L. Liu, Y.-H. Wang, Z.-H. Kang, Y.-G. Li, *Chem. Sci.* **2020**, *11*, 3007.

[18] Y.-J. Liu, P. Shao, M.-Y. Gao, W.-H. Fang, J. Zhang, *Inorg. Chem.* **2020**, *59*, 11442.

[19] B. Zha, C. Li, J. Li, *J. Catal.* **2020**, *382*, 69.

[20] C. Li, B. Zha, J. Li, *J. CO2 Util.* **2020**, *38*, 299.

[21] D. Yang, S. Zuo, H. Yang, X. Wang, *Adv. Energy Mater.* **2021**, *11*, 2100272.

[22] D. Zang, Q. Li, G. Dai, M. Zeng, Y. Huang, Y. Wei, *Appl. Catal. B* **2021**, *281*, 119426.

[23] H. Yang, D. Yang, Y. Zhou, X. Wang, *J. Am. Chem. Soc.* **2021**, *143*, 13721.

[24] J. Ying, B. Zhang, A. Tian, X. Wang, *CrystEngComm* **2021**, *23*, 2572.

[25] H. Yu, J. Ying, Y. Tian, A. Tian, *Inorg. Chem. Commun.* **2021**, *123*, 108338.

[26] B. Zhang, J. Ying, X. Zhang, C. Wang, A. Tian, *New J. Chem.* **2021**, *45*, 13340.

[27] J. Liu, S. Wang, N. Liu, D. Yang, H. Wang, H. Hu, J. Zhuang, X. Wang, *Small* **2021**, *17*, 2006260.

[28] M.-L. Sun, Y.-R. Wang, W.-W. He, R.-L. Zhong, Q.-Z. Liu, S. Xu, J.-M. Xu, X.-L. Han, X. Ge, S.-L. Li, Y.-Q. Lan, A. M. Al-Enizi, A. Nafady, S. Ma, *Small* **2021**, *17*, 2100762.

[29] D. Azaiza-Dabbah, C. Vogt, F. Wang, A. Masip-Sánchez, C. de Graaf, J. M. Poblet, E. Haviv, R. Neumann,

Angew. Chem. Int. Ed. **2022**, *61*, e202112915.
[30] W. Sun, C. Li, H. Zhao, *J. Power Sources* **2022**, *517*, 230665.
[31] Q. Huang, Q. Niu, X.-F. Li, J. Liu, S.-N. Sun, L.-Z. Dong, S.-L. Li, Y.-P. Cai, Y.-Q. Lan, *Sci. Adv.* **2022**, *8*, eadd5598.
[32] S.-Q. Li, L.-F. Dai, Y.-Q. Tian, Y.-X. Yi, J. Yan, C. Liu, *Chem. Commun.* **2023**, *59*, 575.
[33] S. Kuramochi, J. M. Cameron, T. Fukui, K. D. Jones, S. P. Argent, S. Kusaka, R. Shiraishi, Y. Tamaki, T. Yatsu, T. Shiga, O. Ishitani, H. Oshio, G. N. Newton, *Chem. Commun.* **2023**, *59*, 10801.
[34] M.-T. Peng, C. Chen, Y. Zhang, J.-Y. Xu, Y.-L. Teng, B.-X. Dong, *Dalton Trans.* **2023**, *52*, 10737.
[35] W. Sun, Y. Tai, W. Tian, L. Zhou, C. Li, *Electrochim. Acta* **2023**, *445*, 142067.
[36] T. Tubul-Sterin, M. Baranov, G. Gan-Or, N. Leffler, A. Neyman, I. A. Weinstock, *Inorg. Chem.* **2023**, *62*, 1804.
[37] W. Sun, D. Yao, Y. Tai, L. Zhou, W. Tian, M. Yang, C. Li, *J. Colloid Interface Sci.* **2023**, *650*, 121.
[38] W. Sun, L. Zhou, W. Tian, S. Li, M. Yang, C. Li, *Appl. Catal. B* **2024**, *356*, 124206.
[39] K. Kawakami, T. Yabe, F. Amano, K. Yamaguchi, K. Suzuki, *Chem. Sci.* **2024**, *15*, 14829.
[40] L. Shu, C. Li, M.-T. Peng, M.-J. Liu, Y.-L. Teng, B.-X. Dong, *Eur. J. Inorg. Chem.* **2024**, *27*, e202400485.
[41] Y. Zhang, C. Li, L. Shu, Y.-L. Teng, B.-X. Dong, *Inorg. Chem.* **2024**, *63*, 11592.
[42] D. Azaiza-Dabbah, F. Wang, E. Haddad, A. Solé-Daura, R. Carmieli, J. M. Poblet, C. Vogt, R. Neumann, *J. Am. Chem. Soc.* **2024**, *146*, 27871.
[43] J.-J. Liang, Y.-C. Zhang, Z.-H. Chang, Y.-Z. Chen, K.-K. Chen, J.-J. Lu, X.-L. Wang, *New J. Chem.* **2024**, *48*, 1248.
[44] Y. Tai, W. Sun, D. Yao, L. Zhou, W. Tian, H. Yan, C. Li, *Polyoxometalates* **2024**, *3*, 9140051.
[45] P. Zhang, W.-X. Shi, H.-Q. Yin, B.-Q. Sun, Y.-F. Song, T.-B. Lu, Z.-M. Zhang, *ACS Nano* **2025**, *19*, 37206.
[46] C. Liu, L. Song, W. Wu, H. Li, D. Dang, Y. Bai, *Green Chem.* **2025**, *27*, 15607.
[47] W. Sun, C. Li, Y. Tai, L. Zhou, W. Tian, S. Li, *J. Catal.* **2025**, *450*, 116254.
[48] W. Sun, C. Li, L. Zhou, W. Tian, S. Li, M. Yang, *J. Colloid Interface Sci.* **2025**, *697*, 137912.
[49] C. Li, Y. Zhang, L. Shu, Y.-L. Teng, Y.-R. Wang, B.-X. Dong, *Polyoxometalates* **2025**, *4*, 9140097.
[50] K. Xia, T. Yatabe, K. Yonesato, K. Kawakami, S. Kikkawa, S. Yamazoe, K. Yamaguchi, K. Suzuki, *Small* **2026**, *22*, e14728.
